# Comparative Analysis of the Complete Plastomes of *Apostasia wallichii* and *Neuwiedia singapureana* (Apostasioideae) Reveals Different Evolutionary Dynamics of IR/SSC Boundary among Photosynthetic Orchids

**DOI:** 10.3389/fpls.2017.01713

**Published:** 2017-10-04

**Authors:** Zhitao Niu, Jiajia Pan, Shuying Zhu, Ludan Li, Qingyun Xue, Wei Liu, Xiaoyu Ding

**Affiliations:** College of Life Sciences, Nanjing Normal University, Nanjing, China

**Keywords:** Apostasioideae, plastome, hotspots, substitution rates, IR expansion/contraction

## Abstract

Apostasioideae, consists of only two genera, *Apostasia* and *Neuwiedia*, which are mainly distributed in Southeast Asia and northern Australia. The floral structure, taxonomy, biogeography, and genome variation of Apostasioideae have been intensively studied. However, detailed analyses of plastome composition and structure and comparisons with those of other orchid subfamilies have not yet been conducted. Here, the complete plastome sequences of *Apostasia wallichii* and *Neuwiedia singapureana* were sequenced and compared with 43 previously published photosynthetic orchid plastomes to characterize the plastome structure and evolution in the orchids. Unlike many orchid plastomes (e.g., *Paphiopedilum* and *Vanilla*), the plastomes of Apostasioideae contain a full set of 11 functional NADH dehydrogenase (*ndh*) genes. The distribution of repeat sequences and simple sequence repeat elements enhanced the view that the mutation rate of non-coding regions was higher than that of coding regions. The 10 loci—*ndhA* intron, *matK-5′trnK*, *clpP-psbB*, *rps8-rpl14*, *trnT-trnL*, *3′trnK-matK*, *clpP intron*, *psbK-trnK*, *trnS-psbC*, and *ndhF-rpl32*—that had the highest degrees of sequence variability were identified as mutational hotspots for the *Apostasia* plastome. Furthermore, our results revealed that plastid genes exhibited a variable evolution rate within and among different orchid genus. Considering the diversified evolution of both coding and non-coding regions, we suggested that the plastome-wide evolution of orchid species was disproportional. Additionally, the sequences flanking the inverted repeat/small single copy (IR/SSC) junctions of photosynthetic orchid plastomes were categorized into three types according to the presence/absence of *ndh* genes. Different evolutionary dynamics for each of the three IR/SSC types of photosynthetic orchid plastomes were also proposed.

## Introduction

Orchidaceae (orchids) is one of the most diverse and widespread families, comprising approximately 25,000 species in 880 genera (Chase et al., 2003). Their colorful flowers are often found in long sprays and stay fresh for months, giving orchids great ornamental as well as commercial value. According to molecular studies, orchids have been divided into five subfamilies: Apostasioideae, Vanilloideae, Cypripedioideae, Orchidoideae, and Epidendroideae (e.g., Givnish et al., 2015; Kim H.T. et al., 2015). Apostasioideae, the basal-most subfamily, consists of only two genera, *Apostasia* and *Neuwiedia*, with 18 species that are mainly distributed in Southeast Asia and northern Australia (Govaerts et al., 2012). These genera share some apomorphies with other orchids, e.g., enlarged petals (labella), a cylindrical structure of a fused gynoecium and anther (column) and small seeds, but they possess several unique characters, in particular their vessel cells in the root (Stern et al., 1993).

Because of their plesiomorphic floral characters and sister-group to the remaining orchids, members of the Apostasioideae have been intensively studied for their floral structure, taxonomy, biogeography, and genome variation. Kocyan and Endress (2001) analyzed the relationship between Apostasioideae and other orchids by studying their floral structure and development. The phylogenetic relationship within Apostasioideae was also evaluated based on ITS, *trnL-trnF* and *matK* sequences (Kocyan et al., 2004). Jersáková et al. (2013) reported a variable genome size between *Apostasia* and *Neuwiedia*, with a nearly 16-fold range. Kolanowska et al. (2016) assessed the evolution of cold tolerance among Apostasioideae species and proposed that their distribution was affected by climatic change. More recently, the genome sequence of *Apostasia odorata* has been sequenced (Lin et al., 2017). Although numerous studies have been conducted, there still is a lack of information regarding the molecular evolution and species identification of Apostasioideae.

Chloroplasts, responsible for photosynthesis in green plants, are one of the essential organelles in plant cells. Their own genomes (plastomes) vary in size from 59 (Delannoy et al., 2011) to 218 kb (Chumley et al., 2006). The plastome has long been a focus of research in plant molecular evolution and systematics due to its simple structure, highly conserved sequence, and maternal inheritance characteristics (Raubeson and Jansen, 2005). The plastome consists of four parts, including two copies of large inverted repeats (IRs) separated by a large single copy (LSC) and a small single copy (SSC) region (Jansen and Ruhlman, 2012). In general, plastomes are structurally highly conserved across land plants. However, structural rearrangements, gene loss, IR expansion and inversion occur in certain lineages and have been shown to be extremely informative in resolving deep phylogenetic relationships and species identification because they may exhibit less homoplasy than sequence data (Wu et al., 2007; Wang et al., 2008; Downie and Jansen, 2015). Moreover, the complete plastomes are an ideal resource for selecting mutational hotspots (Ahmed et al., 2013; Shaw et al., 2014; Downie and Jansen, 2015). Advances in next-generation sequencing techniques have improved our understanding of orchid plastome organization and evolution (e.g., Kim K. et al., 2015). For example, comparative analyses of orchid plastomes revealed the independent loss of NADH dehydrogenase (*ndh*) genes and diverse patterns of junctions between IR and SSC regions among orchid genera and subfamilies (e.g., Chang et al., 2006; Jheng et al., 2012; Kim H.T. et al., 2015). In addition, a plastome-wide investigation showed diversified hotspots in *Cymbidium* and *Phalaenopsis* (Yang et al., 2013; Shaw et al., 2014). Thus far, however, detailed analyses of apostasioid orchid plastome composition and structure and comparisons with those of other orchid subfamilies have not yet been conducted.

To characterize the plastome structure and evolution in the Apostasioideae subfamily, we sequenced the complete plastome sequences of *Apostasia wallichii* and *Neuwiedia singapureana*, which represent the genera *Apostasia* and *Neuwiedia*, respectively. The genome organization and the gene content and order were compared between the two species and with 45 previously reported photosynthetic orchid plastomes. Our aims were (1) to comparatively study the plastome structure and evolution of *Apostasia* and *Neuwiedia*, (2) to identify more powerful mutational hotspots for Apostasioideae, and (3) to evaluate the evolution of photosynthetic orchid plastomes and the mechanisms that underlie the shifts of their IR boundaries.

## Materials and Methods

### DNA Extraction and Plastome Sequencing

Healthy leaves (2 g) were harvested from individuals of *A. wallichii* and *N. singapureana* grown in the greenhouse of Nanjing Normal University. The total DNA from the leaves was extracted using a Qiagen DNeasy Plant Mini Kit (Qiagen, Germany) based on the manufacturer’s protocol. The quality of the obtained DNA was examined using a NanoDrop 8000 spectrophotometer (Thermo Fisher Scientific, Wilmington, DE, United States). Extracted DNA that met the criteria for analysis (concentration >150 ng/μl, A260/A280 = 1.8–2.0, and A260/A230 > 1.7) was sequenced at 1Gene, Hangzhou (Hangzhou, China), using an Illumina HiSeq 4000 sequencer. The sequencing depth was 3.75 Gb of 150-bp paired-end reads for each species.

### Plastome Assembly, Annotation, and Comparison

The raw sequencing reads were quality trimmed with an error probability of <0.05 and *de novo* assembled using CLC Genomic Workbench 6.0.1 (CLC Bio, Aarhus, Denmark). Contigs of <30× sequencing depths were discarded. Since the remaining contigs may contain the information not only from chloroplast genome, but also from nuclear genome or from mitochondrial genome, these contigs were searched using NCBI Blastn against the plastome sequences of *A. odorata* (NC_030722). Matched contigs with *E*-values of <10^-10^ were designated plastomic contigs. The gaps between plastomic contigs were closed by obtaining amplicons with specific primers and directly sequencing the amplicons. Junctions between the LSC/SSC and IRs were amplified and confirmed by PCR assays. Genes were annotated using DOGMA (Wyman et al., 2004) and tRNAscan-SE 1.21 (Schattner et al., 2005). The exact boundaries of the annotated genes were confirmed by their alignment with their orthologous genes from published orchid plastomes.

The plastome sequences of *A. odorata* (NC_030722), *N. singapureana* (KM 244735) have been published in GenBank of NCBI. However, detailed analyses of plastome composition and structure and comparisons with those of other orchid species have not yet been conducted. Therefore, in this study, they were downloaded and compared with our newly sequenced two plastomes of *A. wallichii* (LC199394) and *N. singapureana* (LC199503) by using the mVISTA software (Frazer et al., 2004). The *N. singapureana* (KM 244735) was used as the reference.

### Repeat Sequence and SSR Element Analysis

The size and location of repeat sequences, including palindromic, reverse and direct repeats, within the newly sequenced plastomes of *A. wallichii* (LC199394) and *N. singapureana* (LC199503) were identified using REPuter software (Kurtz et al., 2001). The repeats were identified according to the following conditions: (1) hamming distance of 3, (2) sequence identity ≥90%, and (3) minimum repeat size ≥30 bp. The simple sequence repeat (SSR) elements were detected using the tool GMATo (Wang et al., 2013), and the criteria of the “Min-length” for mononucleotide SSRs, dinucleotide SSRs, and multi-nucleotide SSRs were set to be greater than 8, 5, and 3 units, respectively.

### Estimation of Sequence Divergence

For mutational hotspot selection, at least two complete plastomes from different species within the study genus should be available (e.g., Ahmed et al., 2013; Shaw et al., 2014). However, only the plastome of *N. singapureana* was sequenced in *Neuwiedia* genus. Therefore, the plastomes of *A. wallichii* and *A. odorata* were used to screen for the most informative regions for the genus *Apostasia*. The sequences of protein-coding genes and non-coding loci, including intergenic spacers and introns, were retrieved from the plastomes of *A. wallichii* and *A. odorata*. Non-coding loci <150 bp were discarded. The sequence alignments were conducted using MUSCLE (Edgar, 2004) implemented in Mega 5.2 (Tamura et al., 2011). The sequences of protein-coding genes were aligned with the Align Codons option using the default parameters. The sequences of non-coding loci were first aligned with the default parameters and then realigned with the “Refining” option. The gaps located at the 5′- and 3′-ends of the alignments were excluded. The yielded alignments were used to count the pairwise nucleotide substitutions and the insertion and deletion (InDel) events using DnaSP v5 (Librado and Rozas, 2009). The sequence variability (SV) was calculated according to the method of Shaw et al. (2005, 2014) and Downie and Jansen (2015): SV% = (the number of nucleotide mutations + the number of InDel events)/(the number of conserved sites + the number of nucleotide mutations + the number of InDel events) × 100.

### Substitution Rates and Positive Selection Analysis

The sequences of 66 protein-coding genes were retrieved from the 45 orchid plastomes and the outgroup plastome of *Lilium longiflorum* (Supplementary Table S1). The sequence alignments of these genes were separately performed and then concatenated to generate a data set with 44,898 characters. The synonymous (ds) and non-synonymous (dn) substitution rates were estimated with the CodeML program of PAML 4.8 (Yang, 2007). The parameters were set to the following: seqtype = 1, runmodel = -2. The SV for each non-coding locus of 10 genera from five orchid subfamilies: Epidendroideae (*Cymbidium*, *Phalaenopsis*, *Masdevallia*, *Dendrobium*, and *Bletilla*), Orchidoideae (*Goodyera*), Cypripedioideae (*Paphiopedilum* and *Cypripedium*), Vanilloideae (*Vanilla*), and Apostasioideae (*Apostasia*) were calculated in Niu et al. (2017) and this study. In order to evaluate the plastome-wide variation of substitution rates among orchid species, the synonymous (ds) and non-synonymous (dn) substitution rates for each protein-coding gene of those 10 genera were estimated in this study.

The selective pressure on the 66 genes from the 45 orchid species was also examined. Firstly, maximum likelihood tree was constructed using RAxML 8.0.2 (Stamatakis, 2014) based on the concatenated data set with a GTRGAMMA model. The genes of *L. longiflorum* were used as outgroup. Secondly, the selective pressure on the 66 genes was analyzed using the site models in the CodeML program. LRT *P*-values were determined for three pairs of site models: M1a vs. M2a, M0 vs. M3, and M7 vs. M8.

### Statistical Analysis

Mesquite v. 3.02 (Maddison and Maddison, 2011) was employed to evaluate the correlation between the shift of IR boundaries and the retained *ndh* gene length. The boundary of IRs that was used for the comparison was a region from the 5′ end of *ycf1* to the junction between the IR and SSC (**Figure 6**). Statistical analyses with Spearman and Mann–Whitney tests were performed using SPSS Statistics 20.0.

## Results

### Sequencing and Plastome Assembly

A total of approximately 3.75 Gb of 150 bp pair-end reads for *A. wallichii* (3.83 Gb) and *N. singapureana* (3.67 Gb) were obtained from the Illumina paired-end sequencing. The *de novo* assembly produced 25,033 contigs for *A. wallichii*, 19,755 contigs for *N. singapureana*. Those contigs were searched against the plastome sequences of *A. odorata* (NC_030722). Then, 52 contigs were obtained with *E*-values <10^-10^ and mean coverage depth >30× for *A. wallichii*, 87 contigs for *N. singapureana*. Among them, five contigs that longer than 18 kb resulting in a nearly complete draft genome for *A. wallichii*. Eleven contigs that longer than 9 kb were used for assembling the plastome of *N. singapureana*. Gaps between contigs (5 gaps in *A. wallichii* plastome and 11 gaps in *N. singapureana* plastome) were closed by obtaining amplicons from PCR procedures. After assembly and gap closure, two complete plastomes were obtained. Furthermore, the four junctions between the LSC/SSC and IRs of each plastomes were also confirmed by PCR amplification.

### Plastome Features of Two Newly Sequenced Orchids

**FIGURE 1:**
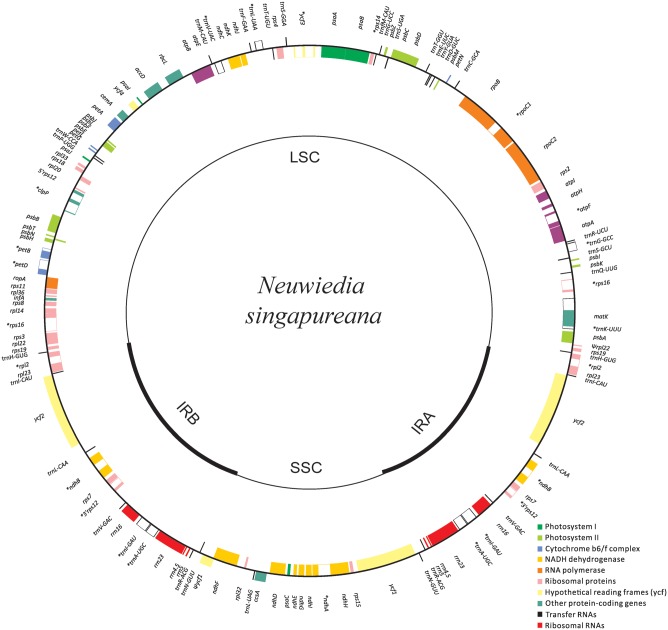
Plastome map of *Neuwiedia singapureana*. The plastome of *Apostasia wallichii* has an identical structure to that of *N. singapureana*, except for an inversion from *trnS-GCU* to *trnS-GGA*. The genes outside and inside the circle are transcribed clockwise and counterclockwise, respectively.

The newly sequenced plastomes of *A. wallichii* (LC199394) and *N. singapureana* (LC199503) were circular and contained 156,126 and 161,068 bp, respectively (**Figure 1**). The plastomes displayed the typical quadripartite structure, consisting of a pair of IRs (26,452–26,991 bp) separated by the LSC (83,035–89,031 bp) and SSC (20,187–18,058 bp) regions (**Table 1**). The gene content of the two plastomes was relatively conserved with identical complements, each containing 79 unique protein-coding genes, 30 unique tRNA genes, and four unique rRNA genes. The overall AT content was 63.93–64.01%, indicating nearly identical levels among the orchid plastomes. The AT content was 66.70–66.79%, 71.26–70.83%, and 56.81–67.14% in the LSC, SSC, and IR regions, respectively (**Table 1**). Notably, the region from *trnS-GCU* to *trnS-GGA* was reversed in the two *Apostasia* plastomes. The four plastomes of Apostasioideae were compared and plotted using mVISTA, with *N. singapureana* (KM 244735) as the reference (**Supplementary Figure S1**). As expected, the SC regions were more divergent than the IR regions. Furthermore, the non-coding regions (intergenic spacers and introns) exhibited higher divergence levels than coding regions.

**Table 1:** Characteristics of the newly sequenced two Apostasioideae plastomes.

Species	Accession	Plastome length (bp)	LSC length (bp)	SSC length (bp)	IR length (bp)	AT content (%)	AT content in LSC (%)	AT content in SSC (%)	AT content in IR (%)
*Apostasia wallichii*	LC199394	156,126	83,035	20,187	26,452	63.93	66.7	71.26	56.81
*Neuwiedia singapureana*	LC199503	161,068	89,031	18,058	26,991	64.01	66.79	70.83	57.14


### Repeat and SSR Analysis

**FIGURE 2:**
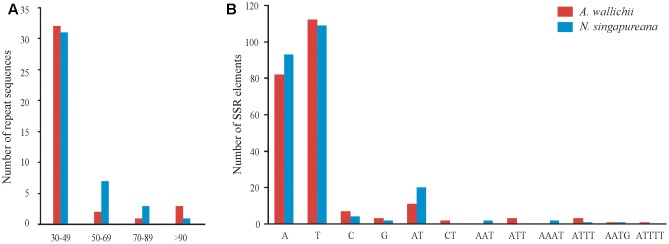
Analysis of repeat sequences and SSR elements in the plastomes of *Apostasia wallichii* and *Neuwiedia singapureana*. **(A)** Number of repeat sequences identified by REPuter. **(B)** Number of SSR elements determined by GMATo.

We employed REPuter to analyze the repeat sequences of the *A. wallichii* and *N. singapureana* plastomes. The total number of repeats was 38 in *A. wallichii* and 42 in *N. singapureana. A. wallichii* contained 15 forward repeats, 18 palindrome repeats, and 5 reverse repeats (**Figure 2A** and Supplementary Table S2). The length of these repeats ranged from 30 to 156 bp, and a copy length of 30–49 bp was most common (32 repeats), followed by >90 bp (three repeats), 50–69 bp (two repeats), and 70–89 bp (one repeat). *N. singapureana* contained 18 forward repeats, 16 palindrome repeats, and 8 reverse repeats. The length of the 42 repeats ranged from 31 to 140 bp, with a copy length of 30 to 49 bp being most common (31 repeats), while >90 bp was the least common (one repeat). In addition, our analyses revealed that the proportions of repeats located in non-coding regions were higher than in coding regions. For example, the proportion of the repeats located in non-coding regions of the *N. singapureana* plastome was 79.76%, while the repeats located in coding regions only accounted for 16.67%.

The SSRs contained in the two plastomes were also counted (**Figure 2B**). The total number of SSRs identified in the *A. wallichii* and *N. singapureana* plastomes was 225 and 234, respectively. Among them, A- and T-mononucleotides were the most abundant SSRs in both plastomes, while the G- or C-repeats were rare. The number of dinucleotide repeats was slightly higher than other repeats, such as tri-, tetra-, and penta-nucleotides. Similar to the repeat sequences, the ratios of SSRs located in non-coding regions were higher than in coding regions (Mann–Whitney two-sided, *P* < 0.05).

### Sequence Divergence of *Apostasia* Plastomes

**FIGURE 3:**
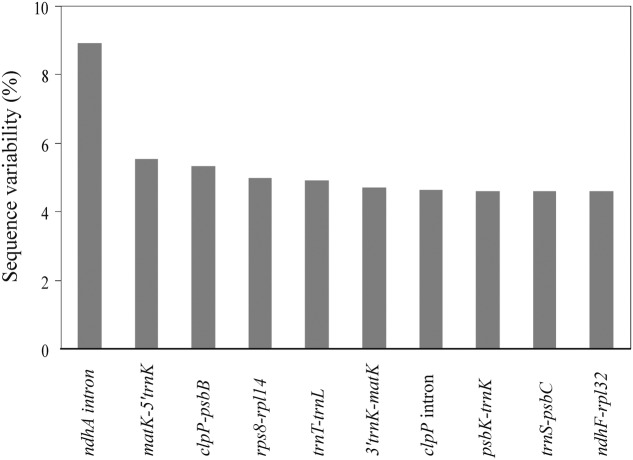
Top 10 syntenic intergenic and intronic loci with the highest sequence variability (%) in *Apostasia* plastomes.

We identified 79 coding genes and 89 syntenic non-coding regions longer than 150 bp in the two *Apostasia* plastomes. The SV for these loci was calculated. The SV value for each coding gene except for a few variable genes (*ycf1*, *rps16*, *rpl20*, and *psaJ*) was below 2.5%, with an average of 0.86% (Supplementary Table S3). The SV in non-coding regions was significantly higher, by approximately 2.6-fold, than that in coding regions (Mann–Whitney two-sided, *P* < 0.05) (Supplementary Table S4). In the non-coding regions, the SV was significantly higher in SC than in IR regions (Mann–Whitney two-sided, *P* < 0.05), which demonstrated that the IR region had a lower sequence divergence than the SC regions. Correlations were significant in the comparisons of (1) SV and GC content (Spearman’s *r* = -0.686, *P* < 0.01), (2) InDels and substitutions (Spearman’s *r* = 0.75, *P* < 0.01), (3) InDels and GC content (Spearman’s *r* = -0.46, *P* < 0.01), and (4) substitutions and GC content (Spearman’s *r* = -0.59, *P* < 0.01). These results indicated that the extent of mutation was correlated with low GC content. Furthermore, the top 10 loci—*ndhA* intron, *matK-5′trnK*, *clpP-psbB*, *rps8-rpl14*, *trnT-trnL*, *3′trnK-matK*, *clpP intron*, *psbK-trnK*, *trnS-psbC*, and *ndhF-rpl32*—listed in **Figure 3** were identified as mutational hotspots for the *Apostasia* plastome that can be used for phylogenetic analyses.

### Non-synonymous (dn) and Synonymous (ds) Substitution Rates among Orchid Species

**FIGURE 4:**
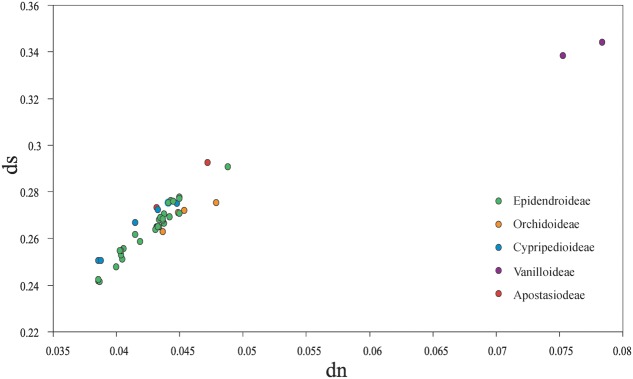
Comparison of non-synonymous (dn) and synonymous (ds) substitution rates among the five orchid subfamilies (Epidendroideae, Orchidoideae, Cypripedioideae, Vanilloideae, and Apostasioideae). The substitutions rates were calculated for the whole plastome by using *Lilium longiflorum* as the reference. Each subfamily is color coded. Of note, the plastomes of Epidendroideae, Orchidoideae, Cypripedioideae, and Apostasioideae showed diversified substitution rates in their plastomic protein-coding sequences.

Among the plastomes of all orchid species, the estimated synonymous (ds) and non-synonymous (dn) substitution rates were 0.2415–0.3439 (substitutions per ds sites) and 0.0387–0.0784 (substitutions per dn site), separately (**Figure 4**). The plastomes of four subfamilies (Epidendroideae, Orchidoideae, Cypripedioideae, and Apostasioideae) showed diversified substitution rates, with dn substitution rates ranging from 0.0387 to 0.0467 and ds substitution rates ranging from 0.2415 to 0.2927, while Vanilloideae (dn: 0.0753–0.0784, ds: 0.3383–0.3439) exhibited significantly higher substitution rates than those of the other orchid subfamilies (Mann–Whitney two-sided, *P* < 0.01).

**FIGURE 5:**
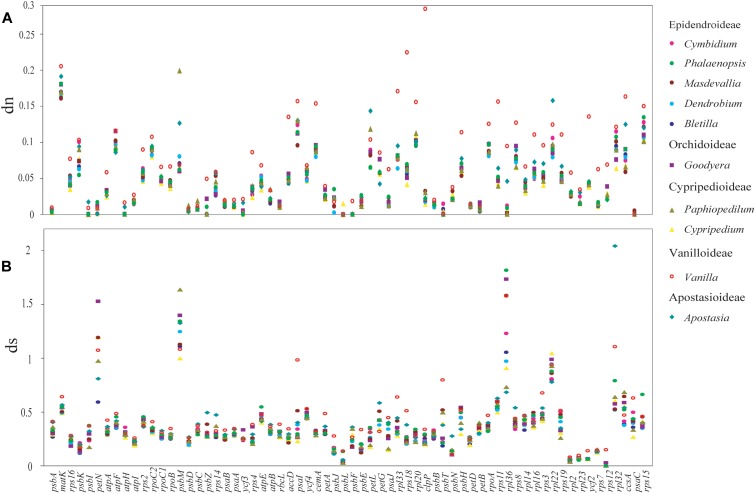
The mean non-synonymous (dn) and synonymous (ds) substitution ratios among 10 genera of orchids. The mean value was calculate from 11 species for *Cymbidium*, two species for *Masdevallia*, three species for *Phalaenopsis*, four species for *Dendrobium*, two species for *Bletilla*, three species for *Goodyera*, two species for *Cypripedium*, two species for *Paphiopedilum*, two species for *Vanilla*, and two species for *Apostasia*. The SD bars for each genus are not indicated. **(A)** Non-synonymous (dn) substitution rates for 66 plastomic protein-coding genes are depicted for 10 genera of orchids. **(B)** Synonymous (ds) substitution rates.

Moreover, the dn and ds rates were also calculated for each protein-coding genes (**Figure 5**). The result displayed a diversified dn and ds values among orchid genera. (1) Genes estimated with the highest dn (ds) rate might not have the highest value for ds (dn). For example, the gene of *rpl22* has the highest dn value in *Apostasia*, whereas, the ds value was the lowest among all the orchid genera. (2) In one orchid genus, the substitutions rates among different genes were variable. For example, in the *Vanilla* plastomes, the dn rate of *clpP* was the highest among all protein-coding genes. The dn rate of *psbB*, the gene adjacent to *clpP*, was lower than most genes. In addition, both of them have showed a low degree of ds rates. (3) The substitution rates of protein-coding genes among different orchid genera were also variable. For example, the gene of *psbC* showed the highest ds rates in the *Apostasia* but lowest in the *Phalaenopsis*. However, the ds rates of *rpl36* exhibited an opposite result. These results indicated that the substitution rates of plastid protein-coding genes were diversified within and among different orchid genus.

Three pairs of site models (M1a vs. M2a, M0 vs. M3, and M7 vs. M8) were used to investigate the possible role of positive selection in driving plastid protein-coding gene evolution in orchid species. The comparison showed that 12 genes related to photosynthetic electron transport and related processes (*psbH*, *atpF*, and *rbcL*), gene expression (*rpoA*, *rpoC1*, *rpoC2*, *rpl2*, *rpl16*, and *rps12*), and other functions (*accD*, *matK*, and *ycf2*) had been subjected to positive selection (LRT M1a vs. M2a, M0 vs. M3, and M7 vs. M8, all *P* < 0.01) (Supplementary Table S5).

### Comparison of Sequences Flanking IR/SSC Junctions among Photosynthetic Orchid Plastomes

**FIGURE 6:**
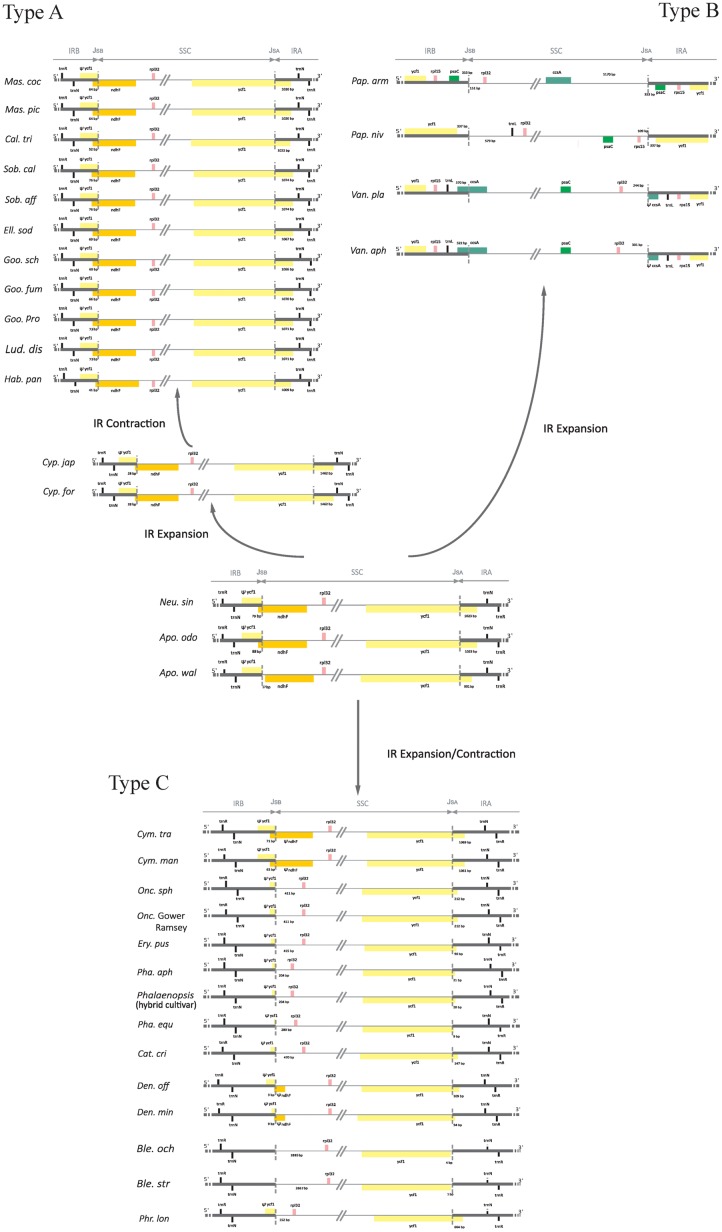
Comparison of IR/SSC boundaries among photosynthetic orchid plastomes. The pseudogenes are denoted by “Ψ.” J_SA_ and J_Sb_ were stand for the junctions between IR_A_/SSC and IR_B_/SSC. The variable length of the 5′ end of *ycf1* to J_SA_ and the intergenic spacer regions adjacent to J_SA_ and J_Sb_ are indicated. The most variable plastomes of *Cymbidium tracyanum* and *Cymbidium mannii* were used to represent the genus *Cymbidium.*

Sequences flanking IR/SSC junctions vary among photosynthetic orchid plastomes (e.g., Yang et al., 2013; Kim H.T. et al., 2015; Lin et al., 2015; Niu et al., 2017). To assess the variability of IR/SSC boundaries, we sampled and categorized 34 available orchid plastomes from 19 genera into three types according to the presence or absence of *ndh* genes. As shown in **Figure 6**, type A, which has all 11 functional *ndh* genes, contains four genera from Epidendroideae (*Calanthe*, *Elleanthus*, *Masdevallia*, and *Sobralia*), three genera from Orchidoideae (*Goodyera*, *Habenaria*, and *Ludisia*), one genus from Cypripedioideae (*Cypripedium*), and the two genera from Apostasioideae (*Apostasia* and *Neuwiedia*). Type B has no *ndh* genes in the SSC region and includes two genera from Cypripedioideae (*Paphiopedilum*) and Vanilloideae (*Vanilla*). Type C, which has independently lost the *ndh* genes in SSC regions, consists of six genera from Epidendroideae (*Cymbidium*, *Cattleya*, *Oncidium*, *Dendrobium*, *Phalaenopsis*, and *Bletilla*) and one genus from Cypripedioideae (*Phragmipedium*). In addition, we determined the degree of IR expansion/contraction based on the length of a region from the 5′ end of the *ycf1* gene to the IR and SSC junction. The IR lengths after expansion/contraction were significantly different among the three types of plastomes (Mann–Whitney two-sided, all *P* < 0.05).

In type A plastomes, the sequences flanking the IR/SSC junctions have similar genic architectures (**Figure 6**). Their junctions of IR_A_/SSC are located approximately 1 kb downstream of the 5′ end of *ycf1*. Conversely, the boundaries of IR/SSC among the four type B species have expanded to variable positions (**Figure 6**). The different IR contraction lengths between the *ndh*-containing and *ndh*-lacking groups (Mann–Whitney two-sided, *P* < 0.05) indicated that the full set of *ndh* genes plays a role in IR/SSC junction stability. Among the orchid species from type C, the junctions of IR/SSC were variable due to the expansion/contraction of IRs (**Figure 6**). For example, the junction of IR_B_/SSC was located at the spacer region adjacent to *rpl32* in most species or to the 3′ end of *ndhF* among *Cymbidium* and *Dendrobium*, while the junctions of IR_A_/SSC were located in *ycf1*, ranging from 9 to 664 bp. The correlation analysis based on the phylogenetic method suggested that the expansion/contraction length of IRs in type C is strongly correlated with the retained *ndh* gene length (**Supplementary Figure S2**, *y* = 0.136*x*, *R*^2^ = 0.847, *P* < 0.05).

## Discussion

### Architecture of Plastid Genomes in Apostasioideae

Complete plastome sequencing and comparative genome analyses have revealed the high conservation of orchid plastomes in size, structure, and gene order and content (e.g., Chang et al., 2006; Jheng et al., 2012; Yang et al., 2013; Kim H.T. et al., 2015; Lin et al., 2015). The structure of Apostasioideae plastomes is similar to that of most orchid plastomes, with the only difference being a large inversion from *trnS-GCU* to *trnS-GGA* in the *Apostasia* plastomes. Plastome rearrangement was also reported for the plastome of *Cypripedium*, which has an inversion from *atpA* to *petG* (Lin et al., 2015). Local disruptions of gene order occur in orchid plastomes because of the loss of *ndh* genes. A character map reconstructed by Kim H.T. et al. (2015) suggested that the *ndh* genes have experienced independent loss in the orchid genera but were present in the common ancestor of orchids. The full set of 11 functional *ndh* genes observed in both of these Apostasioideae plastomes confirmed their results.

Larger repeats are generally rare among angiosperm plastomes and are considered to be more prevalent in those plastomes that have undergone major changes in genome organization (Chumley et al., 2006; Haberle et al., 2008; Weng et al., 2014). Ahmed et al. (2012) reported the association between repeats, InDels and substitution imply that the regions, which have repeat sequences, tend to exhibit higher sequence variation. In the repeat analysis of the newly sequenced plastomes, 38 and 42 repeats of 30 bp or longer were identified in *A. wallichii* and *N. singapureana* plastomes, respectively. Most of these repeats are located in the non-coding regions, including some highly variable regions, such as *5′trnK-matK*, *ndhF-rpl32*, and *rps15-ycf1*. These findings enhance the view that the evolution of non-coding regions was higher than that of coding regions. The repeat sequences have also played an important role in rearrangement in plastomes (Downie and Jansen, 2015; Asaf et al., 2016; Choi et al., 2016; Zhang et al., 2016). However, several repeats occurred in the same genes (*ycf1* and *ycf2*) or genes with similar functions (*pasA*/*psaB* and *trnS-GCU*/*trnS-UGA*/*trnS-GGA*). These repeated elements are similar in number, size, and location to those of other angiosperms whose plastomes are structurally unrearranged (Ruhlman et al., 2006; Yi et al., 2012). Therefore, we suggest that these repeats may have been caused by replication slippage, which generates improper sequence recombination (Palmer, 1991; Downie and Jansen, 2015). Additionally, the SSRs contained in *A. wallichii* and *N. singapureana* were counted. These new resources will be potentially useful for the phylogenetic and population genetics studies in the *Apostasia* and *Neuwiedia* genera.

### Disproportional Plastome-Wide Variation among Orchid Species

Plastid non-coding regions evolve through accumulated nucleotide substitutions and microstructural mutations such as InDels. However, Kelchner (2000) suggested that nucleotide substitutions and InDels would not be randomly distributed throughout plastomes. Morton (1995) and Decker-Walters et al. (2004) proposed that the occurrence of nucleotide substitutions and microstructural mutations depends strongly on sequence context. The extents of SV, InDel events, and substitution rates are related to low GC content, which suggests that mutational hotspots in orchid plastomes are accompanied by biased AT compositions. Indeed, the GC contents of the top 10 mutational hotspots of *Apostasia* plastome were lower than that of most other loci. AT-based mutational hotspots are also observed in taro (Ahmed et al., 2012, 2013), rice (Yamane et al., 2006), and cycad (Wu and Chaw, 2015). AT richness contributes to potentially deleterious recombination and replication errors, which may explain its impact on patterns of substitution and the distribution of InDels.

The top 10 loci—*ndhA* intron, *matK-5′trnK*, *clpP-psbB*, *rps8-rpl14*, *trnT-trnL*, *3′trnK-matK*, *clpP intron*, *psbK-trnK*, *trnS-psbC*, and *ndhF-rpl32*—that have the highest degrees of SV were identified as mutational hotspots for the *Apostasia* plastome. Compared to the mutational hotspots identified in *Cymbidium*, *Phalaenopsis*, and other orchid genera (Yang et al., 2013; Shaw et al., 2014; Niu et al., 2017), the top 10 mutational hotspots are variable among different orchid genera. Although different criteria and methods could affect the identification of the hotspots, our findings in this study suggest that mutational hotspots in orchid plastomes were genus-specific.

Shaw et al. (2014) concluded that a plastid region identified as highly variable in one group might not be consistently the most variable across other group, which suggested that the evolution rates of the non-coding regions among different plant lineages were diversified. In line with this, the current research also revealed that the mutational hotspots were genus-specific among different orchid genera. These results indicated that the evolution rates of the non-coding regions among orchid genera were variable. Moreover, the coding genes also exhibited a diversified evolution rate not only in one orchid specie, but also among different orchid genera. Therefore, we proposed that there is a disproportional plastome-wide variation of substitution rates among orchid species.

Although the substitution rates among protein-coding genes are variable in photosynthetic orchid plastomes, some housekeeping genes related to photosynthesis showed evidence of positive selection pressure. As positive selection pressure would directly affect photosynthetic efficiency, whether it can benefit orchid adaptation is worthy of further investigations.

### Dynamic Evolution of Three IR/SSC Types in Photosynthetic Orchid Plastomes

The expansion and contraction of the border position of the IR can be used to provide relationship evidence in phylogenetic analysis and have been used successfully in resolving relationships of major clades in ferns (Gao et al., 2009), Pinaceae (Lin et al., 2010), Poaceae (Guisinger et al., 2010), Apiaceae (Downie and Jansen, 2015), and many monocots (Wang et al., 2008). However, although overall genomic structures and gene orders are highly conserved, orchid plastomes exhibited obvious differences at the IR/SSC boundaries, which cannot readily be used in a phylogenetic analysis. Moreover, the *ndh* genes in SSC regions have been independently lost across orchid genera (e.g., Kim H.T. et al., 2015; Lin et al., 2015; Niu et al., 2017). The variations in the sequences flanking IR/SSC junctions and the independent loss of *ndh* genes have attracted the intense attention of researchers, leading to the publication of numerous plastomic comparative studies. For example, Luo et al. (2014) suggested dividing the orchid plastomes into four types based on distinct characteristics at the IR/SC junctions. Kim H.T. et al. (2015) proposed that the instability of the IR/SSC junctions in orchid was strongly correlated with the deletion of the *ndhF* gene. However, there is still a lack of information on the mechanism underlying the variations in the sequences flanking the IR/SSC junctions of orchid plastomes.

In the present study, we categorized 34 available photosynthetic orchid plastomes from 19 genera into three types according to the presence of *ndh* genes. The different IR contraction lengths between the plastomes from type A and type B and the correlation between the expansion/contraction length of IRs and the retained *ndh* gene length in type C lead us to conclude that the variations in sequences flanking the IR/SSC junctions of photosynthetic orchid plastomes are associated with the loss of *ndh* genes. Furthermore, we propose different evolutionary dynamics for each of the three IR/SSC types of orchid plastomes. For the plastomes in type A, the border position of the IR and SSC was stable. The IR_A_ and SSC first experienced an expansion from 991 bp in *Apostasia* to 1462 bp in *Cypripedium* and then contracted progressively to downstream of the 5′ end of *ycf1*, while the junction of IR_B_ and SSC was located between 17 bp adjacent to *ndhF* and 76 bp near the 5′ end of *ndhF*. For the plastomes in type B, the boundaries of IR/SSC have expanded to variable positions due to the loss of *ndh* genes. The sequences flanking the IR/SSC junctions vary among the type C plastomes. The correlation analysis based on phylogenetics indicated that the variations in IR/SSC junctions are strongly correlated with the retained *ndh* gene length. However, more molecular data still needs to be collected for intensifying our understanding of the dynamic evolution of sequence variations among IR/SSC junctions.

## Author Contributions

XD designed the study. ZN, SZ, JP, and WL performed the experiments. ZN, JP, LL, and QX analyzed the data. ZN wrote the manuscript.

## Conflict of Interest Statement

The authors declare that the research was conducted in the absence of any commercial or financial relationships that could be construed as a potential conflict of interest.

## Supplementary Material

**FIGURE S1 |** Sequence identity plots among the plastomes of *A. wallichii* (LC199394), *A. odorata* (NC_030722), and *N. singapureana* (LC199503) with the *N. singapureana* (KM 244735) sequence as the reference. The region from *trnS-GCU* to *trnS-GGA* in *A. wallichii* and *A. odorata* plastomes is reversed for comparison.

**FIGURE S2 |** Relationship between the contraction length of IRs and the retained *ndh* gene length in group C. The phylogenetic comparative method indicated that the expansion/contraction of IRs is significantly correlated with the loss of *ndh* genes (*y* = 0.136*x*, *R*^2^ = 0.847, *P* < 0.05).
