# Supplementary figures and images for: Comparative Analysis of the Complete Plastomes of Apostasia wallichii and Neuwiedia singapureana (Apostasioideae) Reveals Different Evolutionary Dynamics of IR/SSC Boundary among Photosynthetic Orchids
Source: Front Plant Sci. 2017 Oct 4;8:1713. doi: 10.3389/fpls.2017.01713 (PMC5632729; doi:10.3389/fpls.2017.01713)

Figure S1

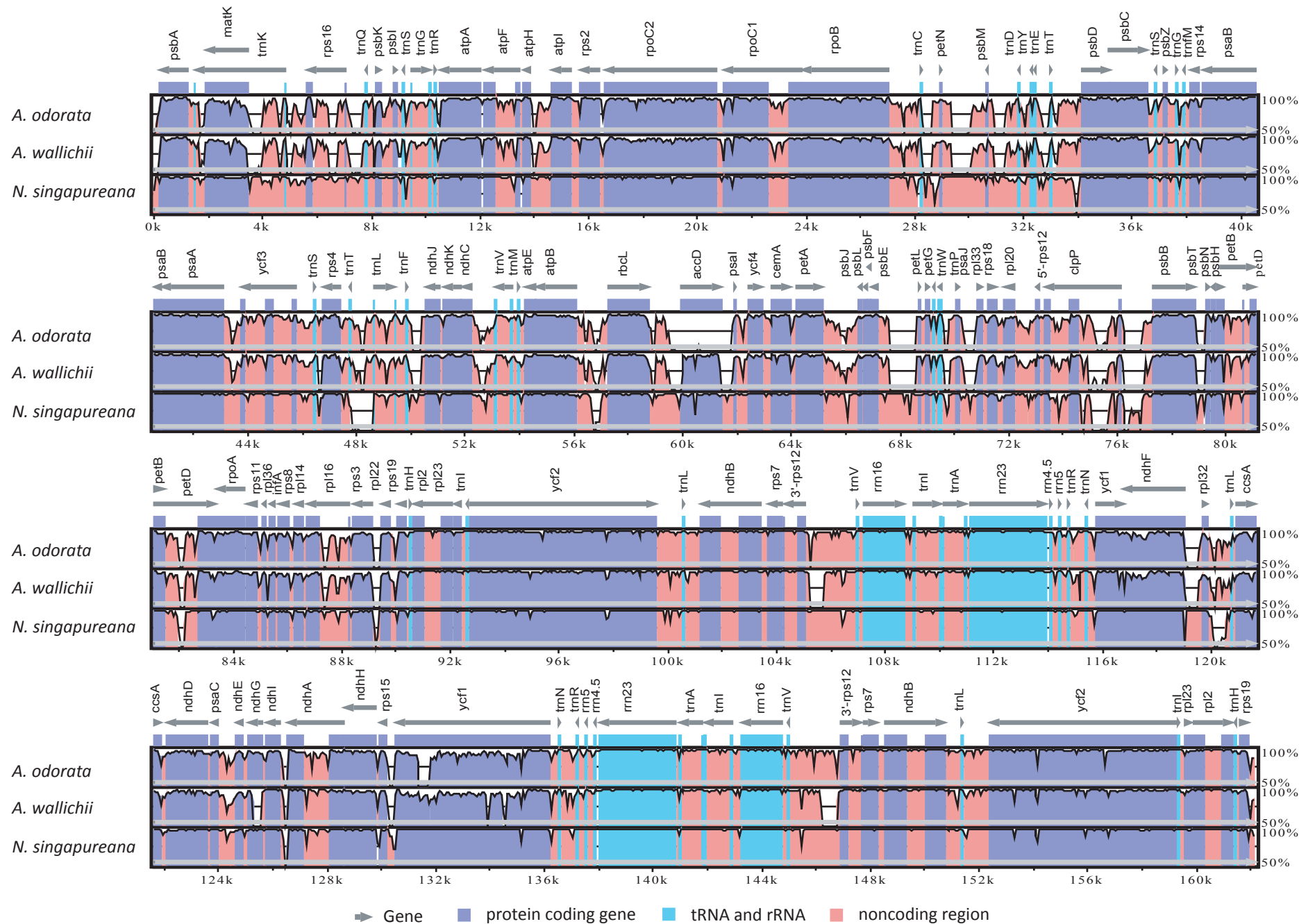

Supplement: Supplementary file 1 [file Image_1.PDF]

Figure S2

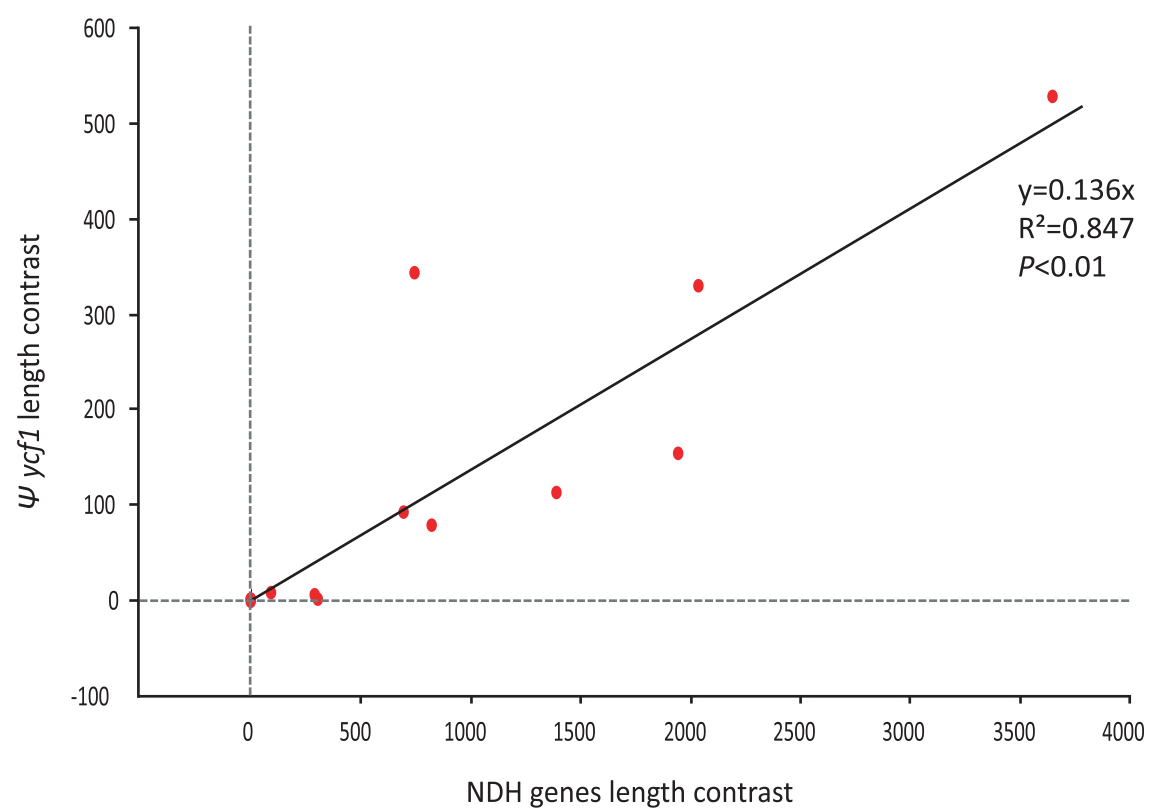

Supplement: Supplementary file 2 [file Image_2.PDF]
