# Supplementary material for: Comparative Analysis of the Complete Plastomes of Apostasia wallichii and Neuwiedia singapureana (Apostasioideae) Reveals Different Evolutionary Dynamics of IR/SSC Boundary among Photosynthetic Orchids
Source: Front Plant Sci. 2017 Oct 4;8:1713. doi: 10.3389/fpls.2017.01713 (PMC5632729; doi:10.3389/fpls.2017.01713)
Supplement: Supplementary file 3 [file Table_1.DOC]

| Table S1 Taxa and their GenBank accession numbers in this study | | | |
| --- | --- | --- | --- |
| Family | Subfamily | Taxon | Accession |
| Orchidaceae | Epidendroideae | *Cymbidium sinense* | NC_021430 |
|  |  | *Cymbidium tortisepalum* | NC_021431 |
|  |  | *Cymbidium tracyanum* | NC_021432 |
|  |  | *Cymbidium mannii* | NC_021433 |
|  |  | *Cymbidium aloifolium* | NC_021429 |
|  |  | *Cymbidium kanran* | NC_029711 |
|  |  | *Cymbidium ensifolium* | NC_028525 |
|  |  | *Cymbidium faberi* | NC_027743 |
|  |  | *Cymbidium goeringii* | NC_028524 |
|  |  | *Cymbidium lancifolium* | NC_029712 |
|  |  | *Cymbidium macrorhizon* | NC_029713 |
|  |  | *Oncidium Gower Ramsey* | NC_014056 |
|  |  | *Oncidium sphacelatum* | NC_028148 |
|  |  | *Erycina pusilla* | NC_018114 |
|  |  | *Cattleya crispata* | NC_026568 |
|  |  | *Masdevallia coccinea* | NC_026541 |
|  |  | *Masdevallia picturata* | NC_026777 |
|  |  | *Phalaenopsis equestris* | NC_017609 |
|  |  | *Phalaenopsis aphrodite* | NC_007499 |
|  |  | *Phalaenopsis* (*hybrid cultivar*) | NC_025593 |
|  |  | *Calanthe triplicata* | NC_024544 |
|  |  | *Dendrobium loddigesii* | LC086479-LC086534 |
|  |  | *Dendrobium officinale* | NC_024019 |
|  |  | *Dendrobium moniliforme* | AB893950 |
|  |  | *Dendrobium huoshanense* | NC_028430 |
|  |  | *Bletilla ochracea* | NC_029483 |
|  |  | *Bletilla striata* | NC_028422 |
|  |  | *Elleanthus sodiroi* | NC_027266 |
|  |  | *Sobralia aff. bouchei* | NC_028209 |
|  |  | *Sobralia callosa* | NC_028147 |
|  | Orchidoideae | *Habenaria pantlingiana* | NC_026775 |
|  |  | *Goodyera schlechtendaliana* | LC085346 |
|  |  | *Goodyera fumata* | NC_026773 |
|  |  | *Goodyera procera* | NC_029363 |
|  |  | *Ludisia discolor* | NC_030540 |
|  | Cypripedioideae | *Cypripedium formosanum* | NC_026772 |
|  |  | *Cypripedium japonicum* | NC_027227 |
|  |  | *Paphiopedilum armeniacum* | LC085347 |
|  |  | *Paphiopedilum niveum* | NC_026776 |
|  |  | *Phragmipedium longifolium* | NC_028149 |
|  | Vanilloideae | *Vanilla aphylla* | LC085348 |
|  |  | *Vanilla planifolia* | NC_026778 |
|  | Apostasiodeae | *Neuwiedia singapureana* | LC199394 |
|  |  | *Apostasia wallichii* | LC199503 |
|  | | *Apostasia odorata* | NC_030722 |
| Liliaceae | | *Lilium longiflorum* | KC968977 |
