# Supplementary material for: Comparative Analysis of the Complete Plastomes of Apostasia wallichii and Neuwiedia singapureana (Apostasioideae) Reveals Different Evolutionary Dynamics of IR/SSC Boundary among Photosynthetic Orchids
Source: Front Plant Sci. 2017 Oct 4;8:1713. doi: 10.3389/fpls.2017.01713 (PMC5632729; doi:10.3389/fpls.2017.01713)
Supplement: Supplementary file 4 [file Table_2.DOC]

| Table S2 Repeat sequence in the plastomes of *A. wallichii* and *N. singapureana* | | | | | | | | | | | |
| --- | --- | --- | --- | --- | --- | --- | --- | --- | --- | --- | --- |
| species | No. | Repeat Type | Length | Position A | Region |  | Locus | Position B | Region |  | Locus |
| *A. wallichii* | 1 | F | 156 | 124183 | SSC | IGS | *rps15-ycf1* | 126819 | SSC | CDS | *ycf1* |
|  | 2 | F | 105 | 123646 | SSC | IGS | *rps15-ycf1* | 126264 | SSC | CDS | *ycf1* |
|  | 3 | F | 97 | 123492 | SSC | IGS | *rps15-ycf1* | 126167 | SSC | CDS | *ycf1* |
|  | 4 | F | 70 | 124339 | SSC | IGS | *rps15-ycf1* | 126969 | SSC | CDS | *ycf1* |
|  | 5 | F | 65 | 123852 | SSC | IGS | *rps15-ycf1* | 126462 | SSC | CDS | *ycf1* |
|  | 6 | P | 50 | 28422 | LSC | IGS | *petN-psbM* | 28422 | LSC | IGS | *petN-psbM* |
|  | 7 | P | 49 | 94357 | IR | IGS | *trnL-ndhB* | 94357 | IR | IGS | *trnL-ndhB* |
|  | 8 | P | 49 | 144755 | IR | IGS | *ndhB-trnL* | 144755 | IR | IGS | *ndhB-trnL* |
|  | 9 | P | 48 | 9861 | LSC | CDS, IGS | *trnR, trnR-atpA* | 9861 | LSC | CDS, IGS | *trnR,trnR-atpA* |
|  | 10 | P | 48 | 28933 | LSC | IGS | *petN-psbM* | 28933 | LSC | IGS | *petN-psbM* |
|  | 11 | F | 48 | 88623 | IR | CDS | *ycf2* | 88644 | IR | CDS | *ycf2* |
|  | 12 | P | 48 | 88644 | IR | CDS | *ycf2* | 150490 | IR | CDS | *ycf2* |
|  | 13 | F | 48 | 150469 | IR | IGS | *trnL-ycf2* | 150490 | IR | IGS | *trnL-ycf2* |
|  | 14 | F | 45 | 123805 | SSC | IGS | *rps15-ycf1* | 126418 | SSC | CDS | *ycf1* |
|  | 15 | R | 44 | 75878 | LSC | intron | *petD* intorn | 75878 | LSC | intron | *petD* intorn |
|  | 16 | F | 44 | 123760 | SSC | IGS | *rps15-ycf1* | 126374 | SSC | CDS | *ycf1* |
|  | 17 | P | 41 | 12976 | LSC | IGS | *atpF-atpH* | 62525 | LSC | IGS | *petA-psbJ* |
|  | 18 | P | 39 | 28432 | LSC | IGS | *petN-psbM* | 28432 | LSC | IGS | *petN-psbM* |
|  | 19 | F | 39 | 43029 | LSC | intron | *ycf3* intron 1 | 98411 | IR | IGS | *3'rps12-trnV* |
|  | 20 | F | 38 | 38081 | LSC | CDS | *psaB* | 40305 | LSC | CDS | *psaA* |
|  | 21 | P | 37 | 123855 | SSC | IGS | *rps15-ycf1* | 123855 | SSC | IGS | *rps15-ycf1* |
|  | 22 | P | 37 | 123855 | SSC | IGS | *rps15-ycf1* | 126465 | SSC | CDS | *ycf1* |
|  | 23 | P | 37 | 126465 | SSC | CDS | *ycf1* | 126465 | SSC | CDS | *ycf1* |
|  | 24 | P | 36 | 31345 | LSC | IGS | *trnT-psbD* | 31345 | LSC | IGS | *trnT-psbD* |
|  | 25 | P | 36 | 41782 | LSC | IGS, CDS | *psaA-ycf3, ycf3* | 41782 | LSC | IGS, CDS | *psaA-ycf3, ycf3* |
|  | 26 | R | 35 | 3352 | LSC | intron | *5'trnK-matK* | 3352 | LSC | intron | *5'trnK-matK* |
|  | 27 | R | 35 | 3368 | LSC | intron | *5'trnK-matK* | 3368 | LSC | intron | *5'trnK-matK* |
|  | 28 | F | 35 | 49939 | LSC | IGS | *ndhC-trnV* | 49955 | LSC | IGS | *ndhC-trnV* |
|  | 29 | P | 34 | 3432 | LSC | intron | *5'trnK-matK* | 3432 | LSC | intron | *5'trnK-matK* |
|  | 30 | F | 34 | 70058 | LSC | intron | *clpP* intron 1 | 70072 | LSC | intron | *clpP* intron 1 |
|  | 31 | P | 33 | 1 | LSC | IGS | *rps19-psbA* | 83137 | IR | IGS | *rpl22-rps19* |
|  | 32 | F | 33 | 88638 | IR | CDS | *ycf2* | 88659 | IR | CDS | *ycf2* |
|  | 33 | P | 33 | 88659 | IR | CDS | *ycf2* | 150490 | IR | CDS | *ycf2* |
|  | 34 | R | 31 | 4235 | LSC | intron | *5'trnK-matK* | 4237 | LSC | intron | *5'trnK-matK* |
|  | 35 | F | 31 | 4887 | LSC | IGS | *trnK-rps16* | 5922 | LSC | intron | *rps16* intron |
|  | 36 | P | 31 | 65018 | LSC | IGS | *petG-trnW* | 65056 | LSC | IGS | *petG-trnW* |
|  | 37 | R | 30 | 45876 | LSC | IGS | *trnT-trnL* | 45879 | LSC | IGS | *trnT-trnL* |
|  | 38 | P | 30 | 70545 | LSC | intron | *clpP* intron 1 | 70545 | LSC | intron | *clpP* intron 1 |
| *N. singapureana* | 1 | F | 140 | 32867 | LSC | IGS | *trnD-trnY* | 33010 | LSC | IGS | *trnD-trnY* |
|  | 2 | P | 89 | 32307 | LSC | IGS | *psbM-trnD* | 68266 | LSC | IGS | *psbE-petL* |
|  | 3 | F | 88 | 4360 | LSC | intron | *5'trnK-matK* | 4448 | LSC | intron | *5'trnK-matK* |
|  | 4 | F | 77 | 73059 | LSC | IGS | *rpl20-5'rps12* | 73136 | LSC | IGS | *rpl20-5'rps12* |
|  | 5 | P | 65 | 118745 | SSC | IGS | *ndhF-rpl32* | 119167 | SSC | IGS | *rpl32-trnL* |
|  | 6 | F | 61 | 3747 | LSC | intron | *5'trnK-matK* | 3799 | LSC | intron | *5'trnK-matK* |
|  | 7 | F | 61 | 3747 | LSC | intron | *5'trnK-matK* | 4152 | LSC | intron | *5'trnK-matK* |
|  | 8 | F | 61 | 3799 | LSC | intron | *5'trnK-matK* | 4100 | LSC | intron | *5'trnK-matK* |
|  | 9 | F | 61 | 4100 | LSC | intron | *5'trnK-matK* | 4152 | LSC | intron | *5'trnK-matK* |
|  | 10 | P | 59 | 1 | LSC | IGS | *rps19-psbA* | 88972 | LSC | CDS | *rpl22* |
|  | 11 | P | 54 | 122690 | SSC | IGS | *ndhD-psaC* | 122690 | SSC | IGS | *ndhD-psaC* |
|  | 12 | P | 49 | 32258 | LSC | IGS | *psbM-trnD* | 68360 | LSC | IGS | *psbE-petL* |
|  | 13 | F | 47 | 33714 | LSC | IGS | *trnE-trnT* | 33762 | LSC | IGS | *trnE-trnT* |
|  | 14 | P | 46 | 31313 | LSC | IGS | *petN-psbM* | 31313 | LSC | IGS | *petN-psbM* |
|  | 15 | P | 44 | 53460 | LSC | IGS | *ndhC-trnV* | 53470 | LSC | IGS | *ndhC-trnV* |
|  | 16 | F | 41 | 63890 | LSC | IGS, CDS | *ycf4-cemA, cemA* | 63930 | LSC | CDS | *cemA* |
|  | 17 | R | 40 | 30581 | LSC | IGS | *petN-psbM* | 30581 | LSC | IGS | *petN-psbM* |
|  | 18 | F | 39 | 45956 | LSC | intron | *ycf3* intron 2 | 104304 | IR | IGS | *3'rps12-trnV* |
|  | 19 | R | 39 | 53466 | LSC | IGS | *ndhC-trnV* | 53466 | LSC | IGS | *ndhC-trnV* |
|  | 20 | P | 39 | 55002 | LSC | IGS, CDS | *trnM-atpE, atpE* | 55002 | LSC | IGS, CDS | *trnM-atpE, atpE* |
|  | 21 | F | 39 | 76025 | LSC | IGS | *clpP-psbB* | 76060 | LSC | IGS | *clpP-psbB* |
|  | 22 | P | 39 | 118771 | SSC | IGS | *ndhF-rpl32* | 119167 | SSC | IGS | *rpl32-trnL* |
|  | 23 | F | 38 | 1771 | LSC | intron | *matK-3'trnK* | 1787 | LSC | intron | *matK-3'trnK* |
|  | 24 | F | 38 | 40954 | LSC | CDS | *psaB* | 43178 | LSC | CDS | *psaA* |
|  | 25 | P | 38 | 130702 | SSC | CDS | *ycf1* | 130702 | SSC | CDS | *ycf1* |
|  | 26 | F | 37 | 78313 | LSC | IGS | *psbB-psbT* | 78348 | LSC | IGS | *psbB-psbT* |
|  | 27 | P | 37 | 130650 | SSC | CDS | *ycf1* | 130650 | SSC | CDS | *ycf1* |
|  | 28 | P | 36 | 53467 | LSC | IGS | *ndhC-trnV* | 53467 | LSC | IGS | *ndhC-trnV* |
|  | 29 | P | 36 | 53468 | LSC | IGS | *ndhC-trnV* | 53468 | LSC | IGS | *ndhC-trnV* |
|  | 30 | R | 36 | 78285 | LSC | IGS | *psbB-psbT* | 78434 | LSC | IGS | *psbB-psbT* |
|  | 31 | F | 36 | 94505 | IR | CDS | *ycf2* | 94526 | IR | CDS | *ycf2* |
|  | 32 | P | 36 | 94505 | IR | CDS | *ycf2* | 155537 | IR | CDS | *ycf2* |
|  | 33 | R | 35 | 53467 | LSC | IGS | *ndhC-trnV* | 53467 | LSC | IGS | *ndhC-trnV* |
|  | 34 | F | 35 | 53467 | LSC | IGS | *ndhC-trnV* | 53469 | LSC | IGS | *ndhC-trnV* |
|  | 35 | R | 35 | 53469 | LSC | IGS | *ndhC-trnV* | 53469 | LSC | IGS | *ndhC-trnV* |
|  | 36 | P | 34 | 53467 | LSC | IGS | *ndhC-trnV* | 53467 | LSC | IGS | *ndhC-trnV* |
|  | 37 | F | 34 | 53468 | LSC | IGS | *ndhC-trnV* | 87230 | LSC | intron | *rpl16 intron* |
|  | 38 | R | 34 | 70116 | LSC | IGS | *trnP-psaJ* | 70124 | LSC | IGS | *trnP-psaJ* |
|  | 39 | F | 32 | 53467 | LSC | IGS | *ndhC-trnV* | 53475 | LSC | IGS | *ndhC-trnV* |
|  | 40 | R | 31 | 7071 | LSC | intron | *rps16* intron | 7071 | LSC | intron | *rps16 intron* |
|  | 41 | P | 31 | 9630 | LSC | CDS | *trnS* | 47416 | LSC | CDS | *trnS* |
|  | 42 | R | 31 | 53466 | LSC | IGS | *ndhC-trnV* | 53466 | LSC | IGS | *ndhC-trnV* |
|  | | | | | | | | | | | |
