# Supplementary material for: Comparative Analysis of the Complete Plastomes of Apostasia wallichii and Neuwiedia singapureana (Apostasioideae) Reveals Different Evolutionary Dynamics of IR/SSC Boundary among Photosynthetic Orchids
Source: Front Plant Sci. 2017 Oct 4;8:1713. doi: 10.3389/fpls.2017.01713 (PMC5632729; doi:10.3389/fpls.2017.01713)
Supplement: Supplementary file 5 [file Table_3.DOC]

| Table S3 Comparisons of 79 protein coding genes from the plastomes of *Apostasia* | | | | |
| --- | --- | --- | --- | --- |
| Gene | aligned length | Substitutions | InDel | Sequence variability (%) |
| *ycf1* | 5703 | 297 | 22 | 6.023414 |
| *rps16* | 315 | 10 | 2 | 4.83871 |
| *rpl20* | 393 | 13 | 2 | 4.076087 |
| *psaJ* | 135 | 4 | 0 | 2.962963 |
| *matK* | 1467 | 27 | 9 | 2.453988 |
| *atpF* | 555 | 12 | 1 | 2.363636 |
| *ycf3* | 546 | 8 | 1 | 1.730769 |
| *infA* | 234 | 4 | 0 | 1.709402 |
| *rpl22* | 363 | 3 | 1 | 1.659751 |
| *rps8* | 396 | 6 | 0 | 1.515152 |
| *ycf4* | 555 | 7 | 0 | 1.261261 |
| *ccsA* | 978 | 12 | 0 | 1.226994 |
| *rps2* | 711 | 8 | 0 | 1.125176 |
| *petN* | 90 | 1 | 0 | 1.111111 |
| *rpl14* | 369 | 4 | 0 | 1.084011 |
| *rps15* | 279 | 3 | 0 | 1.075269 |
| *rps3* | 669 | 5 | 2 | 1.067073 |
| *petL* | 96 | 1 | 0 | 1.041667 |
| *psbK* | 192 | 2 | 0 | 1.041667 |
| *rpl33* | 201 | 2 | 0 | 0.995025 |
| *rps14* | 303 | 3 | 0 | 0.990099 |
| *rps18* | 309 | 3 | 0 | 0.970874 |
| *psaI* | 111 | 1 | 0 | 0.900901 |
| *rpl36* | 114 | 1 | 0 | 0.877193 |
| *rpoC2* | 4164 | 32 | 3 | 0.844798 |
| *rbcL* | 1464 | 10 | 1 | 0.753941 |
| *atpE* | 405 | 3 | 0 | 0.740741 |
| *cemA* | 720 | 3 | 2 | 0.732064 |
| *rps19* | 279 | 2 | 0 | 0.716846 |
| *rpoA* | 1020 | 7 | 0 | 0.686275 |
| *petD* | 483 | 3 | 0 | 0.621118 |
| *rpl32* | 183 | 1 | 0 | 0.546448 |
| *rpoC1* | 2064 | 9 | 2 | 0.537109 |
| *petA* | 963 | 5 | 0 | 0.519211 |
| *rpoB* | 3213 | 16 | 0 | 0.497977 |
| *rps4* | 606 | 3 | 0 | 0.49505 |
| *rpl2* | 819 | 3 | 1 | 0.489596 |
| *rpl16* | 420 | 2 | 0 | 0.47619 |
| *atpB* | 1497 | 7 | 0 | 0.467602 |
| *petB* | 663 | 2 | 1 | 0.46225 |
| *psbH* | 222 | 1 | 0 | 0.45045 |
| *psbE* | 252 | 1 | 0 | 0.396825 |
| *ycf2* | 6921 | 16 | 8 | 0.35263 |
| *atpA* | 1524 | 5 | 0 | 0.328084 |
| *psbB* | 1527 | 5 | 0 | 0.327439 |
| *psbA* | 1062 | 3 | 0 | 0.282486 |
| *psbC* | 1464 | 4 | 0 | 0.273224 |
| *psaA* | 2253 | 6 | 0 | 0.266312 |
| *rps11* | 417 | 1 | 0 | 0.239808 |
| *rps7* | 468 | 1 | 0 | 0.213675 |
| *accD* | 1473 | 3 | 0 | 0.203666 |
| *psbD* | 1062 | 2 | 0 | 0.188324 |
| *psaB* | 2205 | 4 | 0 | 0.181406 |
| *clpP* | 612 | 1 | 0 | 0.163399 |
| *atpI* | 744 | 1 | 0 | 0.134409 |
| *atpH* | 246 | 0 | 0 | 0 |
| *petG* | 114 | 0 | 0 | 0 |
| *psaC* | 246 | 0 | 0 | 0 |
| *psbF* | 120 | 0 | 0 | 0 |
| *psbI* | 111 | 0 | 0 | 0 |
| *psbJ* | 123 | 0 | 0 | 0 |
| *psbL* | 117 | 0 | 0 | 0 |
| *psbM* | 105 | 0 | 0 | 0 |
| *psbN* | 132 | 0 | 0 | 0 |
| *psbT* | 108 | 0 | 0 | 0 |
| *psbZ* | 189 | 0 | 0 | 0 |
| *rpl23* | 282 | 0 | 0 | 0 |
