# Supplementary material for: Comparative Analysis of the Complete Plastomes of Apostasia wallichii and Neuwiedia singapureana (Apostasioideae) Reveals Different Evolutionary Dynamics of IR/SSC Boundary among Photosynthetic Orchids
Source: Front Plant Sci. 2017 Oct 4;8:1713. doi: 10.3389/fpls.2017.01713 (PMC5632729; doi:10.3389/fpls.2017.01713)
Supplement: Supplementary file 6 [file Table_4.DOC]

| Table S4 Comparisons of 89 syntenic loci from the plastomes of *Apostasia* | | | | | | |
| --- | --- | --- | --- | --- | --- | --- |
| Locus | Location | Aligned length | Substitutions | InDel | GC content | Sequence variability (%) |
| *psbK-trnK* | LSC | 270 | 8 | 4 | 0.226236 | 4.615385 |
| *3'trnK-matK* | LSC | 436 | 9 | 9 | 0.222222 | 4.699739 |
| *matK-5'trnK* | LSC | 1348 | 40 | 21 | 0.223776 | 5.525362 |
| *trnK-rps16* | LSC | 780 | 11 | 6 | 0.252026 | 3.695652 |
| *rps16* intron | LSC | 280 | 10 | 0 | 0.3 | 3.571429 |
| *rps16-trnQ* | LSC | 662 | 14 | 6 | 0.24031 | 3.960396 |
| *trnQ-psbK* | LSC | 500 | 4 | 4 | 0.272116 | 2.015113 |
| *psbK-psbI* | LSC | 494 | 10 | 4 | 0.236649 | 3.010753 |
| *trnG-trnS* | LSC | 595 | 11 | 4 | 0.243777 | 2.61324 |
| *atpF* intron | LSC | 825 | 7 | 6 | 0.293178 | 1.608911 |
| *atpI-atpH* | LSC | 750 | 9 | 7 | 0.257338 | 2.216066 |
| *rps2-atpI* | LSC | 288 | 5 | 1 | 0.243478 | 2.083333 |
| *rpoC2-rps2* | LSC | 242 | 5 | 2 | 0.293617 | 3.043478 |
| *rpoC1-rpoC2* | LSC | 230 | 3 | 1 | 0.352668 | 1.980198 |
| *rpoC1* intron | LSC | 791 | 5 | 6 | 0.366712 | 1.587302 |
| *trnC-rpoB* | LSC | 1174 | 10 | 8 | 0.269519 | 1.634877 |
| *petN-trnC* | LSC | 316 | 4 | 2 | 0.259076 | 2.054795 |
| *psbM-petN* | LSC | 1113 | 24 | 13 | 0.222222 | 3.490566 |
| *trnD-psbM* | LSC | 1174 | 10 | 8 | 0.269519 | 1.634877 |
| *trnY-trnD* | LSC | 423 | 5 | 6 | 0.279799 | 2.894737 |
| *trnT-trnE* | LSC | 581 | 7 | 8 | 0.247302 | 2.782931 |
| *psbD-trnT* | LSC | 1084 | 12 | 4 | 0.297335 | 1.510859 |
| *trnS-psbC* | LSC | 310 | 8 | 4 | 0.219081 | 4.615385 |
| *psbZ-trnS* | LSC | 239 | 5 | 4 | 0.330454 | 3.947368 |
| *trnG-psbZ* | LSC | 595 | 11 | 4 | 0.243777 | 2.61324 |
| *trnfM-trnG* | LSC | 181 | 4 | 0 | 0.256906 | 2.209945 |
| *rps14-trnfM* | LSC | 166 | 0 | 1 | 0.326284 | 0.60241 |
| *ycf3-psaA* | LSC | 578 | 7 | 5 | 0.285464 | 2.108963 |
| *ycf3* intron*2* | LSC | 788 | 4 | 4 | 0.315689 | 1.020408 |
| *ycf3* intron*1* | LSC | 782 | 8 | 7 | 0.354817 | 2.054795 |
| *trnG* intron | LSC | 723 | 5 | 2 | 0.306306 | 0.969529 |
| *trnS-ycf3* | LSC | 649 | 9 | 1 | 0.271173 | 1.564945 |
| *rps4-trnT* | LSC | 378 | 7 | 2 | 0.278533 | 2.5 |
| *trnT-trnL* | LSC | 764 | 23 | 10 | 0.245084 | 4.925373 |
| *trnL* intron | LSC | 717 | 7 | 6 | 0.295634 | 1.895044 |
| *trnL-trnF* | LSC | 380 | 3 | 1 | 0.311081 | 1.081081 |
| *trnF-ndhJ* | LSC | 241 | 2 | 3 | 0.295745 | 2.155172 |
| *ndhC-trnV* | LSC | 826 | 8 | 9 | 0.199873 | 2.254642 |
| *rpl20-3'rps12* | LSC | 760 | 5 | 6 | 0.311957 | 1.480485 |
| *trnV* intron | LSC | 595 | 4 | 4 | 0.366185 | 1.365188 |
| *trnV-trnM* | LSC | 164 | 1 | 2 | 0.326087 | 1.875 |
| *trnM-atpE* | LSC | 180 | 3 | 0 | 0.272222 | 1.666667 |
| *atpB-rbcL* | LSC | 1222 | 35 | 12 | 0.225876 | 4.268847 |
| *rbcL-accD* | LSC | 1242 | 27 | 10 | 0.248642 | 3.186908 |
| *psaI-ycf4* | LSC | 523 | 3 | 8 | 0.241107 | 2.21328 |
| *ycf4-cemA* | LSC | 257 | 2 | 0 | 0.264591 | 0.77821 |
| *cemA-petA* | LSC | 239 | 3 | 1 | 0.257862 | 1.67364 |
| *petA-psbJ* | LSC | 1258 | 22 | 11 | 0.26853 | 2.700491 |
| *psbE-petL* | LSC | 524 | 3 | 3 | 0.322612 | 1.188119 |
| *petL-petG* | LSC | 189 | 2 | 1 | 0.288043 | 1.666667 |
| *trnW-trnP* | LSC | 171 | 4 | 1 | 0.354167 | 3.012048 |
| *trnP-psaJ* | LSC | 500 | 4 | 4 | 0.272116 | 2.015113 |
| *psaJ-rpl33* | LSC | 223 | 5 | 1 | 0.268966 | 2.816901 |
| *rpl33-rps18* | LSC | 176 | 1 | 1 | 0.287749 | 1.136364 |
| *rps18-rpl20* | LSC | 256 | 0 | 3 | 0.292089 | 1.25 |
| *clpP* intron*2* | LSC | 753 | 8 | 7 | 0.284652 | 2.046385 |
| *clpP* intron | LSC | 1396 | 31 | 21 | 0.2444 | 4.622222 |
| *clpP-psbB* | LSC | 776 | 11 | 9 | 0.204028 | 5.333333 |
| *psbH-petB* | LSC | 740 | 3 | 5 | 0.299781 | 1.257862 |
| *petB* intron | LSC | 204 | 2 | 1 | 0.37469 | 1.5 |
| *petD* intron | LSC | 1070 | 29 | 12 | 0.282905 | 4.432432 |
| *petD-rpoA* | LSC | 158 | 1 | 1 | 0.269841 | 1.265823 |
| *rps11-rpl36* | LSC | 197 | 1 | 3 | 0.239782 | 2.312139 |
| *rps8-rpl14* | LSC | 282 | 7 | 5 | 0.197292 | 5 |
| *rpl16* intron | LSC | 1044 | 8 | 12 | 0.248392 | 2.022245 |
| *rpl16-rps3* | LSC | 178 | 6 | 1 | 0.281609 | 4.093567 |
| *rpl22-rps19* | LSC | 250 | 4 | 4 | 0.272727 | 4.371585 |
| *rpl2* intron | IR | 668 | 2 | 1 | 0.402558 | 0.453172 |
| *rpl23-trnI* | IR | 157 | 2 | 0 | 0.350318 | 1.273885 |
| *ycf2-trnL* | IR | 1006 | 1 | 4 | 0.420172 | 0.514933 |
| *trnL-ndhB* | IR | 553 | 3 | 2 | 0.360075 | 0.959693 |
| *ndhB* intron | IR | 700 | 2 | 0 | 0.392857 | 0.285714 |
| *ndhB-rps7* | IR | 312 | 0 | 0 | 0.358974 | 0 |
| *rps12* intron | IR | 554 | 1 | 1 | 0.398174 | 0.369004 |
| *3'rps12-trnV* | IR | 2044 | 37 | 17 | 0.396699 | 3.982301 |
| *trnV-rrn16* | IR | 224 | 0 | 1 | 0.461187 | 0.465116 |
| *rrn16-trnI* | IR | 315 | 0 | 2 | 0.503257 | 0.664452 |
| *trnI* intron | IR | 954 | 3 | 3 | 0.493651 | 0.638978 |
| *trnA* intron | IR | 802 | 2 | 2 | 0.507491 | 0.498753 |
| *rrn4.5-rrn5* | IR | 220 | 2 | 0 | 0.465909 | 0.909091 |
| *rrn5-trnR* | IR | 257 | 2 | 3 | 0.40159 | 2.008032 |
| *trnR-trnN* | IR | 546 | 1 | 2 | 0.444874 | 0.612245 |
| *trnN-ycf1* | IR | 344 | 0 | 1 | 0.366366 | 0.309598 |
| *ndhF-rpl32* | SSC | 621 | 18 | 8 | 0.173322 | 4.609929 |
| *rpl32-trnL* | SSC | 966 | 16 | 16 | 0.234066 | 3.678161 |
| *ccsA-ndhD* | SSC | 279 | 2 | 1 | 0.24237 | 1.075269 |
| *psaC-ndhE* | SSC | 741 | 13 | 5 | 0.204181 | 2.575107 |
| *ndhE-ndhG* | SSC | 163 | 1 | 1 | 0.253918 | 1.273885 |
| *ndhA* intron | SSC | 1343 | 70 | 14 | 0.271926 | 8.93617 |
