# Supplementary material for: Comparative Analysis of the Complete Plastomes of Apostasia wallichii and Neuwiedia singapureana (Apostasioideae) Reveals Different Evolutionary Dynamics of IR/SSC Boundary among Photosynthetic Orchids
Source: Front Plant Sci. 2017 Oct 4;8:1713. doi: 10.3389/fpls.2017.01713 (PMC5632729; doi:10.3389/fpls.2017.01713)
Supplement: Supplementary file 7 [file Table_5.DOC]

| Table S5 Parameter estimates and log likelihood values under models of variable ω rates among sites | | | | | | | | | | | | | |
| --- | --- | --- | --- | --- | --- | --- | --- | --- | --- | --- | --- | --- | --- |
| Gene | Model | Parameters | | | | | |  | LRT |  | 2△lnL | P | Positively selected sites |
| *accD* | M0 | *ω* | 0.40926 |  |  |  |  |  |  |  |  |  | 1 K, 2 Q, 8 D, 12 G, 13 L, 20 S, 21 S, 28 H, 44 F, 45 I, 48 G, 53 M, 60 M, 73 I, 77 S, 81 I, 95 Q, 100 P, 109 Q, 165 L, 170 S, 171 N, 221 K, 227 S, 229 T, 233 L, 235 H, 241 L, 247 L, 257 Q, 262 L, 264 L, 266 E |
|  | M1a | *p_0_* | 0.75762 | *p_1_* | 0.24238 |  |  | M1a | vs | M2a | 35.991 | 1.5E-08 |  |
|  | M2a | *p_0_* | 0.76696 | *p_1_* | 0.14947 | *p_2_* | 0.08357 |  |  |  |  |  |  |
|  |  | *ω2* | 1 |  |  |  |  |  |  |  |  |  |  |
|  | M3 | *p_0_* | 0.2935 | *p_1_* | 0.57127 | *p_2_* | 0.13522 | M0 | vs | M3 | 222.698004 | 0 |  |
|  |  | *ω_0_* | 0 | *ω_1_* | 0.28847 | *ω_2_* | 2.4673 |  |  |  |  |  |  |
|  | M7 | *p* | 0.06406 | *q* | 0.07852 |  |  |  |  |  |  |  |  |
|  | M8 | *p_0_* | 0.87113 | *p* | 0.68493 | *q* | 2.51301 | M7 | vs | M8 | 45.842736 | 0 |  |
|  |  | *p_1_* | 0.12887 | *ω* | 2.52287 |  |  |  |  |  |  |  |  |
| *atpA* | M0 | *ω* | 0.11082 |  |  |  |  |  |  |  |  |  |  |
|  | M1a | *p_0_* | 0.90385 | *p_1_* | 0.09615 |  |  | M1a | vs | M2a | 0 | 1 |  |
|  | M2a | *p_0_* | 0.90385 | *p_1_* | 0.06252 | *p_2_* | 0.03363 |  |  |  |  |  |  |
|  |  | *ω2* | 1 |  |  |  |  |  |  |  |  |  |  |
|  | M3 | *p_0_* | 0.85798 | *p_1_* | 0.1394 | *p_2_* | 0.00262 | M0 | vs | M3 | 109.956374 | 0 |  |
|  |  | *ω_0_* | 0.03165 | *ω_1_* | 0.64682 | *ω_2_* | 5.88398 |  |  |  |  |  |  |
|  | M7 | *p* | 0.0431 | *q* | 0.2309 |  |  |  |  |  |  |  |  |
|  | M8 | *p_0_* | 0.98139 | *p* | 0.04682 | *q* | 0.28373 | M7 | vs | M8 | 17.053336 | 0.000198 |  |
|  |  | *p_1_* | 0.01861 | *ω* | 2.08757 |  |  |  |  |  |  |  |  |
| *atpB* | M0 | *ω* | 0.13501 |  |  |  |  |  |  |  |  |  |  |
|  | M1a | *p_0_* | 0.88501 | *p_1_* | 0.11499 |  |  | M1a | vs | M2a | 0.492998 | 0.781532 |  |
|  | M2a | *p_0_* | 0.88672 | *p_1_* | 0.10906 | *p_2_* | 0.00422 |  |  |  |  |  |  |
|  |  | *ω2* | 1 |  |  |  |  |  |  |  |  |  |  |
|  | M3 | *p_0_* | 0.6686 | *p_1_* | 0.28179 | *p_2_* | 0.04961 | M0 | vs | M3 | 141.565948 | 0 |  |
|  |  | *ω_0_* | 0 | *ω_1_* | 0.26661 | *ω_2_* | 1.52353 |  |  |  |  |  |  |
|  | M7 | *p* | 0.03903 | *q* | 0.19455 |  |  |  |  |  |  |  |  |
|  | M8 | *p_0_* | 0.94414 | *p* | 0.04392 | *q* | 0.29013 | M7 | vs | M8 | 23.323994 | 8.62E-06 |  |
|  |  | *p_1_* | 0.05586 | *ω* | 1.44862 |  |  |  |  |  |  |  |  |
| *atpE* | M0 | *ω* | 0.2596 |  |  |  |  |  |  |  |  |  |  |
|  | M1a | *p_0_* | 0.83981 | *p_1_* | 0.16019 |  |  | M1a | vs | M2a | 2.374886 | 0.305 |  |
|  | M2a | *p_0_* | 0.87786 | *p_1_* | 0.09235 | *p_2_* | 0.02979 |  |  |  |  |  |  |
|  |  | *ω2* | 1 |  |  |  |  |  |  |  |  |  |  |
|  | M3 | *p_0_* | 0.58439 | *p_1_* | 0.3789 | *p_2_* | 0.03671 | M0 | vs | M3 | 23.727692 | 9.06E-05 |  |
|  |  | *ω_0_* | 0.08225 | *ω_1_* | 0.41492 | *ω_2_* | 3.03137 |  |  |  |  |  |  |
|  | M7 | *p* | 0.35287 | *q* | 0.84631 |  |  |  |  |  |  |  |  |
|  | M8 | *p_0_* | 0.96296 | *p* | 0.82833 | *q* | 2.84024 | M7 | vs | M8 | 7.304632 | 0.025931 |  |
|  |  | *p_1_* | 0.03704 | *ω* | 3.02187 |  |  |  |  |  |  |  |  |
| *atpF* | M0 | *ω* | 0.39325 |  |  |  |  |  |  |  |  |  | 16 S, 49 L, 50 N |
|  | M1a | *p_0_* | 0.70435 | *p_1_* | 0.29565 |  |  | M1a | vs | M2a | 34.787148 | 2.8E-08 |  |
|  | M2a | *p_0_* | 0.66138 | *p_1_* | 0.31847 | *p_2_* | 0.02014 |  |  |  |  |  |  |
|  |  | *ω2* | 1 |  |  |  |  |  |  |  |  |  |  |
|  | M3 | *p_0_* | 0.65874 | *p_1_* | 0.32103 | *p_2_* | 0.02023 | M0 | vs | M3 | 132.39087 | 0 |  |
|  |  | *ω_0_* | 0.10889 | *ω_1_* | 0.98877 | *ω_2_* | 6.43228 |  |  |  |  |  |  |
|  | M7 | *p* | 0.06891 | *q* | 0.08223 |  |  |  |  |  |  |  |  |
|  | M8 | *p_0_* | 0.97867 | *p* | 0.259 | *q* | 0.42558 | M7 | vs | M8 | 36.194242 | 1.4E-08 |  |
|  |  | *p_1_* | 0.02133 | *ω* | 6.22411 |  |  |  |  |  |  |  |  |
| *atpH* | M0 | *ω* | 0.05823 |  |  |  |  |  |  |  |  |  |  |
|  | M1a | *p_0_* | 0.97752 | *p_1_* | 0.02248 |  |  | M1a | vs | M2a | 0 | 1 |  |
|  | M2a | *p_0_* | 0.97752 | *p_1_* | 0.00894 | *p_2_* | 0.01354 |  |  |  |  |  |  |
|  |  | *ω2* | 1 |  |  |  |  |  |  |  |  |  |  |
|  | M3 | *p_0_* | 0.66453 | *p_1_* | 0.33547 | *p_2_* | 0 | M0 | vs | M3 | 4.3714E+14 | 0.903097 |  |
|  |  | *ω_0_* | 0 | *ω_1_* | 0.18093 | *ω_2_* | 58.80502 |  |  |  |  |  |  |
|  | M7 | *p* | 0.08345 | *q* | 0.72759 |  |  |  |  |  |  |  |  |
|  | M8 | *p_0_* | 0.99999 | *p* | 0.08382 | *q* | 0.73186 | M7 | vs | M8 | 1.90E-04 | 0.999905 |  |
|  |  | *p_1_* | 0.00001 | *ω* | 1 |  |  |  |  |  |  |  |  |
| *atpI* | M0 | *ω* | 0.1292 |  |  |  |  |  |  |  |  |  |  |
|  | M1a | *p_0_* | 0.89637 | *p_1_* | 0.10363 |  |  | M1a | vs | M2a | 1.692462 | 0.429029 |  |
|  | M2a | *p_0_* | 0.90823 | *p_1_* | 0.07933 | *p_2_* | 0.01243 |  |  |  |  |  |  |
|  |  | *ω2* | 1 |  |  |  |  |  |  |  |  |  |  |
|  | M3 | *p_0_* | 0.63882 | *p_1_* | 0.33629 | *p_2_* | 0.02488 | M0 | vs | M3 | 46.32652 | 2E-09 |  |
|  |  | *ω_0_* | 0 | *ω_1_* | 0.28347 | *ω_2_* | 2.62097 |  |  |  |  |  |  |
|  | M7 | *p* | 0.04153 | *q* | 0.21201 |  |  |  |  |  |  |  |  |
|  | M8 | *p_0_* | 0.97562 | *p* | 0.06057 | *q* | 0.37716 | M7 | vs | M8 | 11.481402 | 0.003213 |  |
|  |  | *p_1_* | 0.02438 | *ω* | 2.64435 |  |  |  |  |  |  |  |  |
| *ccsA* | M0 | *ω* | 0.30369 |  |  |  |  |  |  |  |  |  |  |
|  | M1a | *p_0_* | 0.76093 | *p_1_* | 0.23907 |  |  | M1a | vs | M2a | 1.729148 | 0.421231 |  |
|  | M2a | *p_0_* | 0.77161 | *p_1_* | 0.20197 | *p_2_* | 0.02642 |  |  |  |  |  |  |
|  |  | *ω2* | 1 |  |  |  |  |  |  |  |  |  |  |
|  | M3 | *p_0_* | 0.67546 | *p_1_* | 0.28062 | *p_2_* | 0.04392 | M0 | vs | M3 | 36.005758 | 2.89E-07 |  |
|  |  | *ω_0_* | 0.08108 | *ω_1_* | 0.66336 | *ω_2_* | 2.58826 |  |  |  |  |  |  |
|  | M7 | *p* | 0.24104 | *q* | 0.47275 |  |  |  |  |  |  |  |  |
|  | M8 | *p_0_* | 0.91283 | *p* | 0.55975 | *q* | 1.97514 | M7 | vs | M8 | 4.883124 | 0.087025 |  |
|  |  | *p_1_* | 0.08717 | *ω* | 1.87054 |  |  |  |  |  |  |  |  |
| *cemA* | M0 | *ω* | 0.37731 |  |  |  |  |  |  |  |  |  |  |
|  | M1a | *p_0_* | 0.5773 | *p_1_* | 0.4227 |  |  | M1a | vs | M2a | 0 | 1 |  |
|  | M2a | *p_0_* | 0.5773 | *p_1_* | 0.35075 | *p_2_* | 0.07196 |  |  |  |  |  |  |
|  |  | *ω2* | 1 |  |  |  |  |  |  |  |  |  |  |
|  | M3 | *p_0_* | 0.48411 | *p_1_* | 0.5006 | *p_2_* | 0.01529 | M0 | vs | M3 | 68.750736 | 0 |  |
|  |  | *ω_0_* | 0.02737 | *ω_1_* | 0.74559 | *ω_2_* | 3.01775 |  |  |  |  |  |  |
|  | M7 | *p* | 0.08267 | *q* | 0.09564 |  |  |  |  |  |  |  |  |
|  | M8 | *p_0_* | 0.79795 | *p* | 0.26253 | *q* | 0.74062 | M7 | vs | M8 | 0.539224 | 0.763676 |  |
|  |  | *p_1_* | 0.20205 | *ω* | 1.12324 |  |  |  |  |  |  |  |  |
| *clpP* | M0 | *ω* | 0.46584 |  |  |  |  |  |  |  |  |  |  |
|  | M1a | *p_0_* | 0.54476 | *p_1_* | 0.45524 |  |  | M1a | vs | M2a | 2.977586 | 0.225645 |  |
|  | M2a | *p_0_* | 0.5462 | *p_1_* | 0.40845 | *p_2_* | 0.04535 |  |  |  |  |  |  |
|  |  | *ω2* | 1 |  |  |  |  |  |  |  |  |  |  |
|  | M3 | *p_0_* | 0.40561 | *p_1_* | 0.48957 | *p_2_* | 0.10481 | M0 | vs | M3 | 70.576316 | 0 |  |
|  |  | *ω_0_* | 0.052 | *ω_1_* | 0.66901 | *ω_2_* | 2.16237 |  |  |  |  |  |  |
|  | M7 | *p* | 0.07494 | *q* | 0.07982 |  |  |  |  |  |  |  |  |
|  | M8 | *p_0_* | 0.91696 | *p* | 0.38387 | *q* | 0.52444 | M7 | vs | M8 | 5.584796 | 0.061274 |  |
|  |  | *p_1_* | 0.08304 | *ω* | 2.2858 |  |  |  |  |  |  |  |  |
| *matK* | M0 | *ω* | 0.44258 |  |  |  |  |  |  |  |  |  | 2 G, 98 L, 115 N, 117 R, 119 W, 129 F, 139 Q, 164 M, 173 S, 266 N, 272 A, 364 G, 388 I |
|  | M1a | *p_0_* | 0.64643 | *p_1_* | 0.35357 |  |  | M1a | vs | M2a | 59.53642 | 0 |  |
|  | M2a | *p_0_* | 0.61076 | *p_1_* | 0.35621 | *p_2_* | 0.03303 |  |  |  |  |  |  |
|  |  | *ω2* | 1 |  |  |  |  |  |  |  |  |  |  |
|  | M3 | *p_0_* | 0.5494 | *p_1_* | 0.41068 | *p_2_* | 0.03992 | M0 | vs | M3 | 381.937162 | 0 |  |
|  |  | *ω_0_* | 0.09967 | *ω_1_* | 0.81009 | *ω_2_* | 3.51563 |  |  |  |  |  |  |
|  | M7 | *p* | 0.07589 | *q* | 0.08741 |  |  |  |  |  |  |  |  |
|  | M8 | *p_0_* | 0.95857 | *p* | 0.42091 | *q* | 0.63955 | M7 | vs | M8 | 68.179006 | 0 |  |
|  |  | *p_1_* | 0.04143 | *ω* | 3.3986 |  |  |  |  |  |  |  |  |
| *petA* | M0 | *ω* | 0.15773 |  |  |  |  |  |  |  |  |  |  |
|  | M1a | *p_0_* | 0.90291 | *p_1_* | 0.09709 |  |  | M1a | vs | M2a | 3.274584 | 0.194506 |  |
|  | M2a | *p_0_* | 0.93009 | *p_1_* | 0.02468 | *p_2_* | 0.04522 |  |  |  |  |  |  |
|  |  | *ω2* | 1 |  |  |  |  |  |  |  |  |  |  |
|  | M3 | *p_0_* | 0.93669 | *p_1_* | 0.03038 | *p_2_* | 0.03292 | M0 | vs | M3 | 79.231172 | 0 |  |
|  |  | *ω_0_* | 0.08682 | *ω_1_* | 1.60438 | *ω_2_* | 1.6044 |  |  |  |  |  |  |
|  | M7 | *p* | 0.19722 | *q* | 0.81496 |  |  |  |  |  |  |  |  |
|  | M8 | *p_0_* | 0.94878 | *p* | 0.78805 | *q* | 6.60225 | M7 | vs | M8 | 24.372548 | 5.1E-06 |  |
|  |  | *p_1_* | 0.05122 | *ω* | 1.82271 |  |  |  |  |  |  |  |  |
| *petB* | M0 | *ω* | 0.07782 |  |  |  |  |  |  |  |  |  |  |
|  | M1a | *p_0_* | 0.93049 | *p_1_* | 0.06951 |  |  | M1a | vs | M2a | 0.430208 | 0.806458 |  |
|  | M2a | *p_0_* | 0.93501 | *p_1_* | 0.06145 | *p_2_* | 0.00353 |  |  |  |  |  |  |
|  |  | *ω2* | 1 |  |  |  |  |  |  |  |  |  |  |
|  | M3 | *p_0_* | 0.76304 | *p_1_* | 0.22709 | *p_2_* | 0.00987 | M0 | vs | M3 | 37.8836 | 1.18E-07 |  |
|  |  | *ω_0_* | 0 | *ω_1_* | 0.2924 | *ω_2_* | 3.16918 |  |  |  |  |  |  |
|  | M7 | *p* | 0.03914 | *q* | 0.23734 |  |  |  |  |  |  |  |  |
|  | M8 | *p_0_* | 0.98795 | *p* | 0.05102 | *q* | 0.36489 | M7 | vs | M8 | 8.312462 | 0.015666 |  |
|  |  | *p_1_* | 0.01205 | *ω* | 2.83837 |  |  |  |  |  |  |  |  |
| *petD* | M0 | *ω* | 0.0472 |  |  |  |  |  |  |  |  |  |  |
|  | M1a | *p_0_* | 0.96426 | *p_1_* | 0.03574 |  |  | M1a | vs | M2a | 1.410668 | 0.493944 |  |
|  | M2a | *p_0_* | 0.96617 | *p_1_* | 0.02849 | *p_2_* | 0.00533 |  |  |  |  |  |  |
|  |  | *ω2* | 1 |  |  |  |  |  |  |  |  |  |  |
|  | M3 | *p_0_* | 0.95091 | *p_1_* | 0.04271 | *p_2_* | 0.00638 | M0 | vs | M3 | 40.6063 | 3.2E-08 |  |
|  |  | *ω_0_* | 0.00919 | *ω_1_* | 0.57212 | *ω_2_* | 3.84186 |  |  |  |  |  |  |
|  | M7 | *p* | 0.0391 | *q* | 0.28068 |  |  |  |  |  |  |  |  |
|  | M8 | *p_0_* | 0.97139 | *p* | 0.04325 | *q* | 0.54546 | M7 | vs | M8 | 19.17574 | 6.86E-05 |  |
|  |  | *p_1_* | 0.02861 | *ω* | 1.20546 |  |  |  |  |  |  |  |  |
| *petG* | M0 | *ω* | 0.17069 |  |  |  |  |  |  |  |  |  |  |
|  | M1a | *p_0_* | 0.9054 | *p_1_* | 0.0946 |  |  | M1a | vs | M2a | 1.846942 | 0.397138 |  |
|  | M2a | *p_0_* | 0.9621 | *p_1_* | 0 | *p_2_* | 0.0379 |  |  |  |  |  |  |
|  |  | *ω2* | 1 |  |  |  |  |  |  |  |  |  |  |
|  | M3 | *p_0_* | 0.9621 | *p_1_* | 0.02612 | *p_2_* | 0.01179 | M0 | vs | M3 | 12.162092 | 0.016186 |  |
|  |  | *ω_0_* | 0.03121 | *ω_1_* | 3.08326 | *ω_2_* | 3.08326 |  |  |  |  |  |  |
|  | M7 | *p* | 0.03505 | *q* | 0.1573 |  |  |  |  |  |  |  |  |
|  | M8 | *p_0_* | 0.96207 | *p* | 3.3149 | *q* | 99 | M7 | vs | M8 | 5.55114 | 0.062314 |  |
|  |  | *p_1_* | 0.03793 | *ω* | 3.08251 |  |  |  |  |  |  |  |  |
| *petL* | M0 | *ω* | 0.33328 |  |  |  |  |  |  |  |  |  |  |
|  | M1a | *p_0_* | 0.7336 | *p_1_* | 0.2664 |  |  | M1a | vs | M2a | 3.434786 | 0.179534 |  |
|  | M2a | *p_0_* | 0.81383 | *p_1_* | 0.08965 | *p_2_* | 0.09652 |  |  |  |  |  |  |
|  |  | *ω2* | 1 |  |  |  |  |  |  |  |  |  |  |
|  | M3 | *p_0_* | 0.871 | *p_1_* | 0.08022 | *p_2_* | 0.04878 | M0 | vs | M3 | 16.688334 | 0.002222 |  |
|  |  | *ω_0_* | 0.19911 | *ω_1_* | 2.74654 | *ω_2_* | 2.74654 |  |  |  |  |  |  |
|  | M7 | *p* | 0.06988 | *q* | 0.08393 |  |  |  |  |  |  |  |  |
|  | M8 | *p_0_* | 0.8964 | *p* | 1.28133 | *q* | 4.04117 | M7 | vs | M8 | 5.4977 | 0.064001 |  |
|  |  | *p_1_* | 0.1036 | *ω* | 3.15654 |  |  |  |  |  |  |  |  |
| *petN* | M0 | *ω* | 0.06687 |  |  |  |  |  |  |  |  |  |  |
|  | M1a | *p_0_* | 0.99999 | *p_1_* | 0.00001 |  |  | M1a | vs | M2a | 2.60E-05 | 0.999987 |  |
|  | M2a | *p_0_* | 1 | *p_1_* | 0 | *p_2_* | 0 |  |  |  |  |  |  |
|  |  | *ω2* | 1 |  |  |  |  |  |  |  |  |  |  |
|  | M3 | *p_0_* | 0 | *p_1_* | 1 | *p_2_* | 0 | M0 | vs | M3 | 3.00E-05 | 1 |  |
|  |  | *ω_0_* | 0 | *ω_1_* | 0.06687 | *ω_2_* | 4.68601 |  |  |  |  |  |  |
|  | M7 | *p* | 0.22025 | *q* | 2.10459 |  |  |  |  |  |  |  |  |
|  | M8 | *p_0_* | 0.99999 | *p* | 0.22026 | *q* | 2.10473 | M7 | vs | M8 | 1.44E-04 | 0.999928 |  |
|  |  | *p_1_* | 0.00001 | *ω* | 1 |  |  |  |  |  |  |  |  |
| *psaA* | M0 | *ω* | 0.05915 |  |  |  |  |  |  |  |  |  |  |
|  | M1a | *p_0_* | 0.9577 | *p_1_* | 0.0423 |  |  | M1a | vs | M2a | 0.008188 | 0.995914 |  |
|  | M2a | *p_0_* | 0.95873 | *p_1_* | 0.02094 | *p_2_* | 0.02033 |  |  |  |  |  |  |
|  |  | *ω2* | 1 |  |  |  |  |  |  |  |  |  |  |
|  | M3 | *p_0_* | 0.95878 | *p_1_* | 0.03371 | *p_2_* | 0.00751 | M0 | vs | M3 | 56.153708 | 0 |  |
|  |  | *ω_0_* | 0.0247 | *ω_1_* | 1.02899 | *ω_2_* | 1.0307 |  |  |  |  |  |  |
|  | M7 | *p* | 0.04062 | *q* | 0.27661 |  |  |  |  |  |  |  |  |
|  | M8 | *p_0_* | 0.96322 | *p* | 0.46462 | *q* | 13.43057 | M7 | vs | M8 | 20.892976 | 2.91E-05 |  |
|  |  | *p_1_* | 0.03678 | *ω* | 1.08444 |  |  |  |  |  |  |  |  |
| *psaB* | M0 | *ω* | 0.07404 |  |  |  |  |  |  |  |  |  |  |
|  | M1a | *p_0_* | 0.95691 | *p_1_* | 0.04309 |  |  | M1a | vs | M2a | 2.00E-06 | 0.999999 |  |
|  | M2a | *p_0_* | 0.95691 | *p_1_* | 0.02395 | *p_2_* | 0.01915 |  |  |  |  |  |  |
|  |  | *ω2* | 1 |  |  |  |  |  |  |  |  |  |  |
|  | M3 | *p_0_* | 0.71646 | *p_1_* | 0.27684 | *p_2_* | 0.0067 | M0 | vs | M3 | 55.791174 | 0 |  |
|  |  | *ω_0_* | 0 | *ω_1_* | 0.22927 | *ω_2_* | 2.26545 |  |  |  |  |  |  |
|  | M7 | *p* | 0.04238 | *q* | 0.27136 |  |  |  |  |  |  |  |  |
|  | M8 | *p_0_* | 0.99251 | *p* | 0.04609 | *q* | 0.32748 | M7 | vs | M8 | 15.245764 | 0.000489 |  |
|  |  | *p_1_* | 0.00749 | *ω* | 2.16959 |  |  |  |  |  |  |  |  |
| *psaC* | M0 | *ω* | 0.00347 |  |  |  |  |  |  |  |  |  |  |
|  | M1a | *p_0_* | 0.99999 | *p_1_* | 0.00001 |  |  | M1a | vs | M2a | 9.94E-04 | 0.999503 |  |
|  | M2a | *p_0_* | 1 | *p_1_* | 0 | *p_2_* | 0 |  |  |  |  |  |  |
|  |  | *ω2* | 1 |  |  |  |  |  |  |  |  |  |  |
|  | M3 | *p_0_* | 0 | *p_1_* | 1 | *p_2_* | 0 | M0 | vs | M3 | 0 | 1 |  |
|  |  | *ω_0_* | 0.00004 | *ω_1_* | 0.00347 | *ω_2_* | 31.46842 |  |  |  |  |  |  |
|  | M7 | *p* | 0.06139 | *q* | 3.39761 |  |  |  |  |  |  |  |  |
|  | M8 | *p_0_* | 0.98676 | *p* | 0.005 | *q* | 1.92066 | M7 | vs | M8 | 2.069818 | 0.355259 |  |
|  |  | *p_1_* | 0.01324 | *ω* | 1 |  |  |  |  |  |  |  |  |
| *psaI* | M0 | *ω* | 0.28008 |  |  |  |  |  |  |  |  |  |  |
|  | M1a | *p_0_* | 0.7712 | *p_1_* | 0.2288 |  |  | M1a | vs | M2a | 2.181596 | 0.335948 |  |
|  | M2a | *p_0_* | 0.85081 | *p_1_* | 0 | *p_2_* | 0.14919 |  |  |  |  |  |  |
|  |  | *ω2* | 1 |  |  |  |  |  |  |  |  |  |  |
|  | M3 | *p_0_* | 0.27727 | *p_1_* | 0.58107 | *p_2_* | 0.14167 | M0 | vs | M3 | 17.995846 | 0.001236 |  |
|  |  | *ω_0_* | 0.04842 | *ω_1_* | 0.23661 | *ω_2_* | 1.98706 |  |  |  |  |  |  |
|  | M7 | *p* | 0.21988 | *q* | 0.37009 |  |  |  |  |  |  |  |  |
|  | M8 | *p_0_* | 0.85845 | *p* | 1.78301 | *q* | 7.9482 | M7 | vs | M8 | 7.700792 | 0.021271 |  |
|  |  | *p_1_* | 0.14155 | *ω* | 1.98318 |  |  |  |  |  |  |  |  |
| *psaJ* | M0 | *ω* | 0.16194 |  |  |  |  |  |  |  |  |  |  |
|  | M1a | *p_0_* | 0.96048 | *p_1_* | 0.03952 |  |  | M1a | vs | M2a | 0 | 1.00E+00 |  |
|  | M2a | *p_0_* | 0.96048 | *p_1_* | 0.01482 | *p_2_* | 0.0247 |  |  |  |  |  |  |
|  |  | *ω2* | 1 |  |  |  |  |  |  |  |  |  |  |
|  | M3 | *p_0_* | 0.43325 | *p_1_* | 0.00008 | *p_2_* | 0.56667 | M0 | vs | M3 | 1.58785 | 0.810974 |  |
|  |  | *ω_0_* | 0 | *ω_1_* | 0.2921 | *ω_2_* | 0.2921 |  |  |  |  |  |  |
|  | M7 | *p* | 0.49894 | *q* | 2.22375 |  |  |  |  |  |  |  |  |
|  | M8 | *p_0_* | 0.99999 | *p* | 0.49895 | *q* | 2.22386 | M7 | vs | M8 | 8.60E-05 | 0.999957 |  |
|  |  | *p_1_* | 0.00001 | *ω* | 1 |  |  |  |  |  |  |  |  |
| *psbA* | M0 | *ω* | 0.03405 |  |  |  |  |  |  |  |  |  |  |
|  | M1a | *p_0_* | 0.97645 | *p_1_* | 0.02355 |  |  | M1a | vs | M2a | 2.923728 | 0.231804 |  |
|  | M2a | *p_0_* | 0.97892 | *p_1_* | 0.01503 | *p_2_* | 0.00605 |  |  |  |  |  |  |
|  |  | *ω2* | 1 |  |  |  |  |  |  |  |  |  |  |
|  | M3 | *p_0_* | 0.98297 | *p_1_* | 0.01703 | *p_2_* | 0 | M0 | vs | M3 | 94.305272 | 0 |  |
|  |  | *ω_0_* | 0.01231 | *ω_1_* | 1.62185 | *ω_2_* | 262.1444 |  |  |  |  |  |  |
|  | M7 | *p* | 0.0392 | *q* | 0.31212 |  |  |  |  |  |  |  |  |
|  | M8 | *p_0_* | 0.98393 | *p* | 0.0455 | *q* | 0.64599 | M7 | vs | M8 | 56.844192 | 0 |  |
|  |  | *p_1_* | 0.01607 | *ω* | 1.6944 |  |  |  |  |  |  |  |  |
| *psbB* | M0 | *ω* | 0.09681 |  |  |  |  |  |  |  |  |  |  |
|  | M1a | *p_0_* | 0.91731 | *p_1_* | 0.08269 |  |  | M1a | vs | M2a | 2.40E-05 | 0.999988 |  |
|  | M2a | *p_0_* | 0.91731 | *p_1_* | 0.04562 | *p_2_* | 0.03707 |  |  |  |  |  |  |
|  |  | *ω2* | 1 |  |  |  |  |  |  |  |  |  |  |
|  | M3 | *p_0_* | 0.70578 | *p_1_* | 0.24823 | *p_2_* | 0.04599 | M0 | vs | M3 | 89.7129 | 0 |  |
|  |  | *ω_0_* | 0 | *ω_1_* | 0.18532 | *ω_2_* | 1.4139 |  |  |  |  |  |  |
|  | M7 | *p* | 0.03872 | *q* | 0.21438 |  |  |  |  |  |  |  |  |
|  | M8 | *p_0_* | 0.94936 | *p* | 0.04431 | *q* | 0.35486 | M7 | vs | M8 | 23.278762 | 0 |  |
|  |  | *p_1_* | 0.05064 | *ω* | 1.35102 |  |  |  |  |  |  |  |  |
| *psbC* | M0 | *ω* | 0.0516 |  |  |  |  |  |  |  |  |  |  |
|  | M1a | *p_0_* | 0.97892 | *p_1_* | 0.02108 |  |  | M1a | vs | M2a | 0.029169826 | 1 |  |
|  | M2a | *p_0_* | 0.97892 | *p_1_* | 0.02107 | *p_2_* | 0.00001 |  |  |  |  |  |  |
|  |  | *ω2* | 1 |  |  |  |  |  |  |  |  |  |  |
|  | M3 | *p_0_* | 0.89196 | *p_1_* | 0.10804 | *p_2_* | 0 | M0 | vs | M3 | 10.77847 | 0 |  |
|  |  | *ω_0_* | 0.01897 | *ω_1_* | 0.34001 | *ω_2_* | 24.7748 |  |  |  |  |  |  |
|  | M7 | *p* | 0.04455 | *q* | 0.33446 |  |  |  |  |  |  |  |  |
|  | M8 | *p_0_* | 0.99457 | *p* | 0.04781 | *q* | 0.38667 | M7 | vs | M8 | 1.305646 | 0.520574 |  |
|  |  | *p_1_* | 0.00543 | *ω* | 1.46588 |  |  |  |  |  |  |  |  |
| *psbD* | M0 | *ω* | 0.04767 |  |  |  |  |  |  |  |  |  |  |
|  | M1a | *p_0_* | 0.97857 | *p_1_* | 0.02143 |  |  | M1a | vs | M2a | 9.60E-05 | 0.999952 |  |
|  | M2a | *p_0_* | 0.97857 | *p_1_* | 0.02143 | *p_2_* | 0 |  |  |  |  |  |  |
|  |  | *ω2* | 1 |  |  |  |  |  |  |  |  |  |  |
|  | M3 | *p_0_* | 0.77697 | *p_1_* | 0.22303 | *p_2_* | 0 | M0 | vs | M3 | 8.632638 | 0.070967 |  |
|  |  | *ω_0_* | 0 | *ω_1_* | 0.22402 | *ω_2_* | 27.15352 |  |  |  |  |  |  |
|  | M7 | *p* | 0.02033 | *q* | 0.12534 |  |  |  |  |  |  |  |  |
|  | M8 | *p_0_* | 0.99999 | *p* | 0.03439 | *q* | 0.24286 | M7 | vs | M8 | 5.68E-04 | 0.999716 |  |
|  |  | *p_1_* | 0.00001 | *ω* | 1 |  |  |  |  |  |  |  |  |
| *psbE* | M0 | *ω* | 0.08426 |  |  |  |  |  |  |  |  |  |  |
|  | M1a | *p_0_* | 0.95737 | *p_1_* | 0.04263 |  |  | M1a | vs | M2a | 1.222062 | 0.542791 |  |
|  | M2a | *p_0_* | 0.97428 | *p_1_* | 0 | *p_2_* | 0.02572 |  |  |  |  |  |  |
|  |  | *ω2* | 1 |  |  |  |  |  |  |  |  |  |  |
|  | M3 | *p_0_* | 0.07977 | *p_1_* | 0.89452 | *p_2_* | 0.02572 | M0 | vs | M3 | 13.239186 | 0.010164 |  |
|  |  | *ω_0_* | 0.04875 | *ω_1_* | 0.04875 | *ω_2_* | 2.07206 |  |  |  |  |  |  |
|  | M7 | *p* | 0.0433 | *q* | 0.26477 |  |  |  |  |  |  |  |  |
|  | M8 | *p_0_* | 0.97498 | *p* | 5.2588 | *q* | 99 | M7 | vs | M8 | 6.539766 | 0.038011 |  |
|  |  | *p_1_* | 0.02502 | *ω* | 2.10372 |  |  |  |  |  |  |  |  |
| *psbF* | M0 | *ω* | 0.06074 |  |  |  |  |  |  |  |  |  |  |
|  | M1a | *p_0_* | 0.9444 | *p_1_* | 0.0556 |  |  | M1a | vs | M2a | 6.00E-05 | 0.99997 |  |
|  | M2a | *p_0_* | 0.9444 | *p_1_* | 0.0247 | *p_2_* | 0.0309 |  |  |  |  |  |  |
|  |  | *ω2* | 1 |  |  |  |  |  |  |  |  |  |  |
|  | M3 | *p_0_* | 0.32682 | *p_1_* | 0.54878 | *p_2_* | 0.1244 | M0 | vs | M3 | 2.696422 | 0.609841 |  |
|  |  | *ω_0_* | 0 | *ω_1_* | 0 | *ω_2_* | 0.46245 |  |  |  |  |  |  |
|  | M7 | *p* | 0.03992 | *q* | 0.27365 |  |  |  |  |  |  |  |  |
|  | M8 | *p_0_* | 0.94815 | *p* | 0.04165 | *q* | 0.47995 | M7 | vs | M8 | 0.431592 | 0.8059 |  |
|  |  | *p_1_* | 0.05185 | *ω* | 1 |  |  |  |  |  |  |  | 16 S, 18 G, 45 I, 72 I |
| *psbH* | M0 | *ω* | 0.28869 |  |  |  |  |  |  |  |  |  |  |
|  | M1a | *p_0_* | 0.8419 | *p_1_* | 0.1581 |  |  | M1a | vs | M2a | 15.498124 | 0.000431 |  |
|  | M2a | *p_0_* | 0.85666 | *p_1_* | 0.09174 | *p_2_* | 0.0516 |  |  |  |  |  |  |
|  |  | *ω2* | 1 |  |  |  |  |  |  |  |  |  |  |
|  | M3 | *p_0_* | 0.61335 | *p_1_* | 0.33136 | *p_2_* | 0.05529 | M0 | vs | M3 | 64.801966 | 0 |  |
|  |  | *ω_0_* | 0.04433 | *ω_1_* | 0.39109 | *ω_2_* | 4.27166 |  |  |  |  |  |  |
|  | M7 | *p* | 0.06241 | *q* | 0.08102 |  |  |  |  |  |  |  |  |
|  | M8 | *p_0_* | 0.94463 | *p* | 0.43769 | *q* | 1.94658 | M7 | vs | M8 | 24.522082 | 4.73E-06 |  |
|  |  | *p_1_* | 0.05537 | *ω* | 4.27017 |  |  |  |  |  |  |  |  |
| *psbI* | M0 | *ω* | 0.05882 |  |  |  |  |  |  |  |  |  |  |
|  | M1a | *p_0_* | 0.99999 | *p_1_* | 0.00001 |  |  | M1a | vs | M2a | 8.40E-05 | 0.999958 |  |
|  | M2a | *p_0_* | 1 | *p_1_* | 0 | *p_2_* | 0 |  |  |  |  |  |  |
|  |  | *ω2* | 1 |  |  |  |  |  |  |  |  |  |  |
|  | M3 | *p_0_* | 0 | *p_1_* | 1 | *p_2_* | 0 | M0 | vs | M3 | 0.672092 | 0.954725 |  |
|  |  | *ω_0_* | 0 | *ω_1_* | 0.05882 | *ω_2_* | 13.44466 |  |  |  |  |  |  |
|  | M7 | *p* | 0.04283 | *q* | 0.30669 |  |  |  |  |  |  |  |  |
|  | M8 | *p_0_* | 0.99999 | *p* | 0.04253 | *q* | 0.30389 | M7 | vs | M8 | 0.6716 | 0.714766 |  |
|  |  | *p_1_* | 0.00001 | *ω* | 2.11599 |  |  |  |  |  |  |  |  |
| *psbJ* | M0 | *ω* | 0.15789 |  |  |  |  |  |  |  |  |  |  |
|  | M1a | *p_0_* | 0.85708 | *p_1_* | 0.14292 |  |  | M1a | vs | M2a | 0.569458 | 0.752218 |  |
|  | M2a | *p_0_* | 0.87261 | *p_1_* | 0.03209 | *p_2_* | 0.0953 |  |  |  |  |  |  |
|  |  | *ω2* | 1 |  |  |  |  |  |  |  |  |  |  |
|  | M3 | *p_0_* | 0.8326 | *p_1_* | 0.07844 | *p_2_* | 0.08896 | M0 | vs | M3 | 21.029984 | 0.000312 |  |
|  |  | *ω_0_* | 0.01477 | *ω_1_* | 0.43679 | *ω_2_* | 1.597 |  |  |  |  |  |  |
|  | M7 | *p* | 0.03174 | *q* | 0.13471 |  |  |  |  |  |  |  |  |
|  | M8 | *p_0_* | 0.88422 | *p* | 0.05378 | *q* | 0.58612 | M7 | vs | M8 | 4.554508 | 0.102565 |  |
|  |  | *p_1_* | 0.11578 | *ω* | 1.43717 |  |  |  |  |  |  |  |  |
| *psbK* | M0 | *ω* | 0.39161 |  |  |  |  |  |  |  |  |  |  |
|  | M1a | *p_0_* | 0.63087 | *p_1_* | 0.36913 |  |  | M1a | vs | M2a | 0.00E+00 | 1 |  |
|  | M2a | *p_0_* | 0.63087 | *p_1_* | 0.21582 | *p_2_* | 0.15331 |  |  |  |  |  |  |
|  |  | *ω2* | 1 |  |  |  |  |  |  |  |  |  |  |
|  | M3 | *p_0_* | 0.62509 | *p_1_* | 0.30482 | *p_2_* | 0.0701 | M0 | vs | M3 | 26.816374 | 2.17E-05 |  |
|  |  | *ω_0_* | 0.08197 | *ω_1_* | 0.97256 | *ω_2_* | 0.97256 |  |  |  |  |  |  |
|  | M7 | *p* | 0.07781 | *q* | 0.09518 |  |  |  |  |  |  |  |  |
|  | M8 | *p_0_* | 0.65668 | *p* | 0.7051 | *q* | 5.43718 | M7 | vs | M8 | 0.068728 | 0.96622 |  |
|  |  | *p_1_* | 0.34332 | *ω* | 1.01734 |  |  |  |  |  |  |  |  |
| *psbL* | M0 | *ω* | 0.0524 |  |  |  |  |  |  |  |  |  |  |
|  | M1a | *p_0_* | 0.95617 | *p_1_* | 0.04383 |  |  | M1a | vs | M2a | 0.01831 | 0.990887 |  |
|  | M2a | *p_0_* | 0.95897 | *p_1_* | 0 | *p_2_* | 0.04103 |  |  |  |  |  |  |
|  |  | *ω2* | 1 |  |  |  |  |  |  |  |  |  |  |
|  | M3 | *p_0_* | 0.92573 | *p_1_* | 0.03324 | *p_2_* | 0.04103 | M0 | vs | M3 | 4.92362 | 0.295225 |  |
|  |  | *ω_0_* | 0 | *ω_1_* | 0 | *ω_2_* | 1.15602 |  |  |  |  |  |  |
|  | M7 | *p* | 0.03803 | *q* | 0.26833 |  |  |  |  |  |  |  |  |
|  | M8 | *p_0_* | 0.95897 | *p* | 0.00687 | *q* | 2.59574 | M7 | vs | M8 | 2.773588 | 0.249875 |  |
|  |  | *p_1_* | 0.04103 | *ω* | 1.15602 |  |  |  |  |  |  |  |  |
| *psbM* | M0 | *ω* | 0.45784 |  |  |  |  |  |  |  |  |  |  |
|  | M1a | *p_0_* | 0.52561 | *p_1_* | 0.47439 |  |  | M1a | vs | M2a | 0.40644 | 0.816099 |  |
|  | M2a | *p_0_* | 0.59925 | *p_1_* | 0 | *p_2_* | 0.40075 |  |  |  |  |  |  |
|  |  | *ω2* | 1 |  |  |  |  |  |  |  |  |  |  |
|  | M3 | *p_0_* | 0.59925 | *p_1_* | 0.2584 | *p_2_* | 0.14235 | M0 | vs | M3 | 11.090124 | 0.02557 |  |
|  |  | *ω_0_* | 0.1062 | *ω_1_* | 1.3671 | *ω_2_* | 1.3671 |  |  |  |  |  |  |
|  | M7 | *p* | 0.07257 | *q* | 0.07836 |  |  |  |  |  |  |  |  |
|  | M8 | *p_0_* | 0.60012 | *p* | 12.0445 | *q* | 99 | M7 | vs | M8 | 1.247036 | 0.536055 |  |
|  |  | *p_1_* | 0.39988 | *ω* | 1.36836 |  |  |  |  |  |  |  |  |
| *psbN* | M0 | *ω* | 0.13623 |  |  |  |  |  |  |  |  |  |  |
|  | M1a | *p_0_* | 0.93449 | *p_1_* | 0.06551 |  |  | M1a | vs | M2a | 0.711576 | 0.700621 |  |
|  | M2a | *p_0_* | 0.97157 | *p_1_* | 0 | *p_2_* | 0.02843 |  |  |  |  |  |  |
|  |  | *ω2* | 1 |  |  |  |  |  |  |  |  |  |  |
|  | M3 | *p_0_* | 0.97157 | *p_1_* | 0.00785 | *p_2_* | 0.02058 | M0 | vs | M3 | 4.816334 | 0.306667 |  |
|  |  | *ω_0_* | 0.08638 | *ω_1_* | 2.58095 | *ω_2_* | 2.58095 |  |  |  |  |  |  |
|  | M7 | *p* | 0.11439 | *q* | 0.57328 |  |  |  |  |  |  |  |  |
|  | M8 | *p_0_* | 0.97163 | *p* | 9.53388 | *q* | 99 | M7 | vs | M8 | 2.028004 | 0.362764 |  |
|  |  | *p_1_* | 0.02837 | *ω* | 2.5809 |  |  |  |  |  |  |  |  |
| *psbT* | M0 | *ω* | 0.03606 |  |  |  |  |  |  |  |  |  |  |
|  | M1a | *p_0_* | 0.99999 | *p_1_* | 0.00001 |  |  | M1a | vs | M2a | 4.60E-04 | 0.99977 |  |
|  | M2a | *p_0_* | 1 | *p_1_* | 0 | *p_2_* | 0 |  |  |  |  |  |  |
|  |  | *ω2* | 1 |  |  |  |  |  |  |  |  |  |  |
|  | M3 | *p_0_* | 0.66664 | *p_1_* | 0.33336 | *p_2_* | 0 | M0 | vs | M3 | 1.209812 | 0.876481 |  |
|  |  | *ω_0_* | 0 | *ω_1_* | 0.11895 | *ω_2_* | 167.0191 |  |  |  |  |  |  |
|  | M7 | *p* | 0.02636 | *q* | 0.18142 |  |  |  |  |  |  |  |  |
|  | M8 | *p_0_* | 0.99999 | *p* | 0.04445 | *q* | 0.37432 | M7 | vs | M8 | 6.34E-04 | 0.999683 |  |
|  |  | *p_1_* | 0.00001 | *ω* | 2.0123 |  |  |  |  |  |  |  |  |
| *psbZ* | M0 | *ω* | 0.16863 |  |  |  |  |  |  |  |  |  |  |
|  | M1a | *p_0_* | 0.81679 | *p_1_* | 0.18321 |  |  | M1a | vs | M2a | 0 | 1 |  |
|  | M2a | *p_0_* | 0.81679 | *p_1_* | 0.10131 | *p_2_* | 0.0819 |  |  |  |  |  |  |
|  |  | *ω2* | 1 |  |  |  |  |  |  |  |  |  |  |
|  | M3 | *p_0_* | 0.6318 | *p_1_* | 0.29867 | *p_2_* | 0.06952 | M0 | vs | M3 | 11.029744 | 0.026232 |  |
|  |  | *ω_0_* | 0 | *ω_1_* | 0.34196 | *ω_2_* | 1.32523 |  |  |  |  |  |  |
|  | M7 | *p* | 0.03973 | *q* | 0.18525 |  |  |  |  |  |  |  |  |
|  | M8 | *p_0_* | 0.93095 | *p* | 0.03965 | *q* | 0.22498 | M7 | vs | M8 | 0.404492 | 0.816894 |  |
|  |  | *p_1_* | 0.06905 | *ω* | 1.34767 |  |  |  |  |  |  |  |  |
| *rbcL* | M0 | *ω* | 0.11432 |  |  |  |  |  |  |  |  |  | 23 T, 78 A, 131 P, 208 L, 211 A, 214 L, 215 F, 219 A, 240 I, 251 V, 315 I, 317 A, 329 E, 330 M, 342 F, 352 F, 364 I, 432 D, 438 S, 450 I, 453 E, 455 T, 457 D, 459 D, 460 P |
|  | M1a | *p_0_* | 0.90346 | *p_1_* | 0.09654 |  |  | M1a | vs | M2a | 16.579942 | 0.000251 |  |
|  | M2a | *p_0_* | 0.90889 | *p_1_* | 0.07082 | *p_2_* | 0.02029 |  |  |  |  |  |  |
|  |  | *ω2* | 1 |  |  |  |  |  |  |  |  |  |  |
|  | M3 | *p_0_* | 0.87528 | *p_1_* | 0.0921 | *p_2_* | 0.03262 | M0 | vs | M3 | 315.676924 | 0 |  |
|  |  | *ω_0_* | 0 | *ω_1_* | 0.53699 | *ω_2_* | 2.54625 |  |  |  |  |  |  |
|  | M7 | *p* | 0.02569 | *q* | 0.11607 |  |  |  |  |  |  |  |  |
|  | M8 | *p_0_* | 0.93646 | *p* | 0.03672 | *q* | 0.36263 | M7 | vs | M8 | 104.934532 | 0 |  |
|  |  | *p_1_* | 0.06354 | *ω* | 1.71108 |  |  |  |  |  |  |  |  |
| *rpl2* | M0 | *ω* | 0.69017 |  |  |  |  |  |  |  |  |  | 1 I, 9 P, 12 R, 14 G, 15 A, 16 V, 24 P, 25 R, 26 K, 30 Y, 32 Q, 33 H, 34 R, 41 A, 60 K, 67 E, 69 G, 93 G, 102 H, 106 A, 113 V, 116 T, 117 E |
|  | M1a | *p_0_* | 0.574 | *p_1_* | 0.426 |  |  | M1a | vs | M2a | 9.323704 | 0.009449 |  |
|  | M2a | *p_0_* | 0.83097 | *p_1_* | 0.07931 | *p_2_* | 0.08972 |  |  |  |  |  |  |
|  |  | *ω2* | 1 |  |  |  |  |  |  |  |  |  |  |
|  | M3 | *p_0_* | 0.84469 | *p_1_* | 0.06589 | *p_2_* | 0.08942 | M0 | vs | M3 | 21.301354 | 0.000276 |  |
|  |  | *ω_0_* | 0.25371 | *ω_1_* | 1.10501 | *ω_2_* | 5.95932 |  |  |  |  |  |  |
|  | M7 | *p* | 0.04297 | *q* | 0.05483 |  |  |  |  |  |  |  |  |
|  | M8 | *p_0_* | 0.71735 | *p* | 0.005 | *q* | 1.79752 | M7 | vs | M8 | 7.7387 | 0.020872 |  |
|  |  | *p_1_* | 0.28265 | *ω* | 2.80284 |  |  |  |  |  |  |  |  |
| *rpl14* | M0 | *ω* | 0.18314 |  |  |  |  |  |  |  |  |  |  |
|  | M1a | *p_0_* | 0.84076 | *p_1_* | 0.15924 |  |  | M1a | vs | M2a | 0 | 1 |  |
|  | M2a | *p_0_* | 0.84076 | *p_1_* | 0.0614 | *p_2_* | 0.09784 |  |  |  |  |  |  |
|  |  | *ω2* | 1 |  |  |  |  |  |  |  |  |  |  |
|  | M3 | *p_0_* | 0.83752 | *p_1_* | 0.16248 | *p_2_* | 0 | M0 | vs | M3 | 37.922386 | 1.16E-07 |  |
|  |  | *ω_0_* | 0.0685 | *ω_1_* | 0.97257 | *ω_2_* | 22.69075 |  |  |  |  |  |  |
|  | M7 | *p* | 0.21995 | *q* | 0.73107 |  |  |  |  |  |  |  |  |
|  | M8 | *p_0_* | 0.84211 | *p* | 7.63557 | *q* | 99 | M7 | vs | M8 | 4.935042 | 0.084795 |  |
|  |  | *p_1_* | 0.15789 | *ω* | 1 |  |  |  |  |  |  |  |  |
| *rpl16* | M0 | *ω* | 0.27161 |  |  |  |  |  |  |  |  |  | 100 K, 104 C, 113 V, 115 S |
|  | M1a | *p_0_* | 0.764 | *p_1_* | 0.236 |  |  | M1a | vs | M2a | 11.89793 | 0.002609 |  |
|  | M2a | *p_0_* | 0.74784 | *p_1_* | 0.22316 | *p_2_* | 0.029 |  |  |  |  |  |  |
|  |  | *ω2* | 1 |  |  |  |  |  |  |  |  |  |  |
|  | M3 | *p_0_* | 0.60754 | *p_1_* | 0.35329 | *p_2_* | 0.03917 | M0 | vs | M3 | 105.924312 | 0 |  |
|  |  | *ω_0_* | 0.02661 | *ω_1_* | 0.53367 | *ω_2_* | 3.09023 |  |  |  |  |  |  |
|  | M7 | *p* | 0.16512 | *q* | 0.31186 |  |  |  |  |  |  |  |  |
|  | M8 | *p_0_* | 0.96138 | *p* | 0.2453 | *q* | 0.83451 | M7 | vs | M8 | 19.851892 | 4.89E-05 |  |
|  |  | *p_1_* | 0.03862 | *ω* | 3.1044 |  |  |  |  |  |  |  |  |
| *rpl20* | M0 | *ω* | 0.33671 |  |  |  |  |  |  |  |  |  |  |
|  | M1a | *p_0_* | 0.74575 | *p_1_* | 0.25425 |  |  | M1a | vs | M2a | 0.053428 | 0.97364 |  |
|  | M2a | *p_0_* | 0.75394 | *p_1_* | 0 | *p_2_* | 0.24606 |  |  |  |  |  |  |
|  |  | *ω2* | 1 |  |  |  |  |  |  |  |  |  |  |
|  | M3 | *p_0_* | 0.75394 | *p_1_* | 0.17733 | *p_2_* | 0.06873 | M0 | vs | M3 | 36.912534 | 1.88E-07 |  |
|  |  | *ω_0_* | 0.14848 | *ω_1_* | 1.05042 | *ω_2_* | 1.05042 |  |  |  |  |  |  |
|  | M7 | *p* | 0.51569 | *q* | 0.92939 |  |  |  |  |  |  |  |  |
|  | M8 | *p_0_* | 0.75649 | *p* | 17.65224 | *q* | 99 | M7 | vs | M8 | 5.430106 | 0.066201 |  |
|  |  | *p_1_* | 0.24351 | *ω* | 1.0555 |  |  |  |  |  |  |  |  |
| *rpl22* | M0 | *ω* | 0.17825 |  |  |  |  |  |  |  |  |  |  |
|  | M1a | *p_0_* | 0.81766 | *p_1_* | 0.18234 |  |  | M1a | vs | M2a | 0.735576 | 0.692264 |  |
|  | M2a | *p_0_* | 0.81911 | *p_1_* | 0.14903 | *p_2_* | 0.03186 |  |  |  |  |  |  |
|  |  | *ω2* | 1 |  |  |  |  |  |  |  |  |  |  |
|  | M3 | *p_0_* | 0.44577 | *p_1_* | 0.42834 | *p_2_* | 0.12589 | M0 | vs | M3 | 89.550714 | 0 |  |
|  |  | *ω_0_* | 0 | *ω_1_* | 0.19604 | *ω_2_* | 1.38388 |  |  |  |  |  |  |
|  | M7 | *p* | 0.16604 | *q* | 0.32141 |  |  |  |  |  |  |  |  |
|  | M8 | *p_0_* | 0.87951 | *p* | 0.34542 | *q* | 2.59674 | M7 | vs | M8 | 9.931454 | 0.006973 |  |
|  |  | *p_1_* | 0.12049 | *ω* | 1.42199 |  |  |  |  |  |  |  |  |
| *rpl23* | M0 | *ω* | 0.36968 |  |  |  |  |  |  |  |  |  |  |
|  | M1a | *p_0_* | 0.79477 | *p_1_* | 0.20523 |  |  | M1a | vs | M2a | 0.653944 | 0.721104 |  |
|  | M2a | *p_0_* | 0.96278 | *p_1_* | 0 | *p_2_* | 0.03722 |  |  |  |  |  |  |
|  |  | *ω2* | 1 |  |  |  |  |  |  |  |  |  |  |
|  | M3 | *p_0_* | 0.15292 | *p_1_* | 0.80985 | *p_2_* | 0.03723 | M0 | vs | M3 | 2.336736 | 0.674089 |  |
|  |  | *ω_0_* | 0.27589 | *ω_1_* | 0.27589 | *ω_2_* | 3.09528 |  |  |  |  |  |  |
|  | M7 | *p* | 0.31428 | *q* | 0.53453 |  |  |  |  |  |  |  |  |
|  | M8 | *p_0_* | 0.96303 | *p* | 37.95219 | *q* | 99 | M7 | vs | M8 | 0.968726 | 0.61609 |  |
|  |  | *p_1_* | 0.03697 | *ω* | 3.10092 |  |  |  |  |  |  |  |  |
| *rpl32* | M0 | *ω* | 0.19358 |  |  |  |  |  |  |  |  |  |  |
|  | M1a | *p_0_* | 0.81042 | *p_1_* | 0.18958 |  |  | M1a | vs | M2a | 0 | 1 |  |
|  | M2a | *p_0_* | 0.81042 | *p_1_* | 0.10463 | *p_2_* | 0.08495 |  |  |  |  |  |  |
|  |  | *ω2* | 1 |  |  |  |  |  |  |  |  |  |  |
|  | M3 | *p_0_* | 0.43814 | *p_1_* | 0.46955 | *p_2_* | 0.09231 | M0 | vs | M3 | 28.270466 | 1.1E-05 |  |
|  |  | *ω_0_* | 0 | *ω_1_* | 0.26422 | *ω_2_* | 1.32107 |  |  |  |  |  |  |
|  | M7 | *p* | 0.27531 | *q* | 0.80369 |  |  |  |  |  |  |  |  |
|  | M8 | *p_0_* | 0.91133 | *p* | 0.35277 | *q* | 1.86135 | M7 | vs | M8 | 2.53333 | 0.28177 |  |
|  |  | *p_1_* | 0.08867 | *ω* | 1.31471 |  |  |  |  |  |  |  |  |
| *rpl33* | M0 | *ω* | 0.26256 |  |  |  |  |  |  |  |  |  |  |
|  | M1a | *p_0_* | 0.79026 | *p_1_* | 0.20974 |  |  | M1a | vs | M2a | 3.517674 | 0.172245 |  |
|  | M2a | *p_0_* | 0.81666 | *p_1_* | 0 | *p_2_* | 0.18334 |  |  |  |  |  |  |
|  |  | *ω2* | 1 |  |  |  |  |  |  |  |  |  |  |
|  | M3 | *p_0_* | 0.81666 | *p_1_* | 0.10882 | *p_2_* | 0.07452 | M0 | vs | M3 | 61.12641 | 0 |  |
|  |  | *ω_0_* | 0.10157 | *ω_1_* | 1.62045 | *ω_2_* | 1.62045 |  |  |  |  |  |  |
|  | M7 | *p* | 0.06608 | *q* | 0.08469 |  |  |  |  |  |  |  |  |
|  | M8 | *p_0_* | 0.81761 | *p* | 11.42513 | *q* | 98.96694 | M7 | vs | M8 | 10.4449 | 0.005394 |  |
|  |  | *p_1_* | 0.18239 | *ω* | 1.62642 |  |  |  |  |  |  |  |  |
| *rpl36* | M0 | *ω* | 0.18446 |  |  |  |  |  |  |  |  |  |  |
|  | M1a | *p_0_* | 0.75166 | *p_1_* | 0.24834 |  |  | M1a | vs | M2a | 0 | 1 |  |
|  | M2a | *p_0_* | 0.75166 | *p_1_* | 0.09814 | *p_2_* | 0.1502 |  |  |  |  |  |  |
|  |  | *ω2* | 1 |  |  |  |  |  |  |  |  |  |  |
|  | M3 | *p_0_* | 0.54688 | *p_1_* | 0.07627 | *p_2_* | 0.37685 | M0 | vs | M3 | 6.107176 | 0.191286 |  |
|  |  | *ω_0_* | 0 | *ω_1_* | 0.5162 | *ω_2_* | 0.5162 |  |  |  |  |  |  |
|  | M7 | *p* | 0.21623 | *q* | 0.67718 |  |  |  |  |  |  |  |  |
|  | M8 | *p_0_* | 0.99999 | *p* | 0.21622 | *q* | 0.67719 | M7 | vs | M8 | 8.00E-06 | 0.999996 |  |
|  |  | *p_1_* | 0.00001 | *ω* | 1 |  |  |  |  |  |  |  |  |
| *rpoA* | M0 | *ω* | 0.33199 |  |  |  |  |  |  |  |  |  | 105 D, 115 Y, 145 L, 158 H, 164 E, 166 H, 253 L, 258 G, 275 T, 301 M, 311 Q, 314 G |
|  | M1a | *p_0_* | 0.72677 | *p_1_* | 0.27323 |  |  | M1a | vs | M2a | 8.599638 | 0.013571 |  |
|  | M2a | *p_0_* | 0.72786 | *p_1_* | 0.24539 | *p_2_* | 0.02675 |  |  |  |  |  |  |
|  |  | *ω2* | 1 |  |  |  |  |  |  |  |  |  |  |
|  | M3 | *p_0_* | 0.66081 | *p_1_* | 0.29891 | *p_2_* | 0.04028 | M0 | vs | M3 | 134.64031 | 0 |  |
|  |  | *ω_0_* | 0.08821 | *ω_1_* | 0.75561 | *ω_2_* | 2.8527 |  |  |  |  |  |  |
|  | M7 | *p* | 0.15672 | *q* | 0.2382 |  |  |  |  |  |  |  |  |
|  | M8 | *p_0_* | 0.95106 | *p* | 0.38046 | *q* | 0.92184 | M7 | vs | M8 | 15.106504 | 0.000524 |  |
|  |  | *p_1_* | 0.04894 | *ω* | 2.65164 |  |  |  |  |  |  |  |  |
| *rpoB* | M0 | *ω* | 0.20533 |  |  |  |  |  |  |  |  |  |  |
|  | M1a | *p_0_* | 0.83566 | *p_1_* | 0.16434 |  |  | M1a | vs | M2a | 2.763252 | 0.25117 |  |
|  | M2a | *p_0_* | 0.84022 | *p_1_* | 0.15248 | *p_2_* | 0.0073 |  |  |  |  |  |  |
|  |  | *ω2* | 1 |  |  |  |  |  |  |  |  |  |  |
|  | M3 | *p_0_* | 0.74046 | *p_1_* | 0.23316 | *p_2_* | 0.02638 | M0 | vs | M3 | 224.559064 | 0 |  |
|  |  | *ω_0_* | 0.05321 | *ω_1_* | 0.5975 | *ω_2_* | 2.20443 |  |  |  |  |  |  |
|  | M7 | *p* | 0.19617 | *q* | 0.61664 |  |  |  |  |  |  |  |  |
|  | M8 | *p_0_* | 0.9507 | *p* | 0.29703 | *q* | 1.37395 | M7 | vs | M8 | 23.671456 | 7.24E-06 |  |
|  |  | *p_1_* | 0.0493 | *ω* | 1.75032 |  |  |  |  |  |  |  |  |
| *rpoC1* | M0 | *ω* | 0.1979 |  |  |  |  |  |  |  |  |  | 21 M, 76 G, 79 T, 146 A, 200 S, 249 L, 257 W, 422 S, 541 L, 561 Y, 571 P, 574 C, 630 R, 666 R |
|  | M1a | *p_0_* | 0.8378 | *p_1_* | 0.1622 |  |  | M1a | vs | M2a | 9.547876 | 0.008447 |  |
|  | M2a | *p_0_* | 0.83911 | *p_1_* | 0.15307 | *p_2_* | 0.00782 |  |  |  |  |  |  |
|  |  | *ω2* | 1 |  |  |  |  |  |  |  |  |  |  |
|  | M3 | *p_0_* | 0.74806 | *p_1_* | 0.23162 | *p_2_* | 0.02032 | M0 | vs | M3 | 211.857514 | 0 |  |
|  |  | *ω_0_* | 0.03921 | *ω_1_* | 0.60071 | *ω_2_* | 2.84262 |  |  |  |  |  |  |
|  | M7 | *p* | 0.16566 | *q* | 0.54955 |  |  |  |  |  |  |  |  |
|  | M8 | *p_0_* | 0.97063 | *p* | 0.19789 | *q* | 0.90524 | M7 | vs | M8 | 32.544488 | 8.6E-08 |  |
|  |  | *p_1_* | 0.02937 | *ω* | 2.43387 |  |  |  |  |  |  |  |  |
| *rpoC2* | M0 | *ω* | 0.32145 |  |  |  |  |  |  |  |  |  | 19 I, 44 T, 231 Q, 233 W, 279 A, 394 I, 424 A, 427 S, 436 Q, 453 G, 481 C, 486 G, 504 L, 520 L, 527 R, 528 T, 537 T, 562 K, 607 Y, 623 G, 624 A, 629 S, 635 Q, 651 S, 702 V, 718 E, 722 S, 728 L, 767 F, 790 G, 817 K, 850 S, 863 L, 872 I, 881 A, 885 S, 890 K, 891 G, 899 R, 907 I, 916 R, 918 G, 928 A, 935 P, 938 D, 939 L, 951 V, 953 L, 970 Y, 991 R, 1021 A, 1022 W, 1038 L, 1203 L, 1302 L, 1304 I, 1310 Y |
|  | M1a | *p_0_* | 0.74096 | *p_1_* | 0.25904 |  |  | M1a | vs | M2a | 22.708828 | 1.17E-05 |  |
|  | M2a | *p_0_* | 0.74125 | *p_1_* | 0.24436 | *p_2_* | 0.01439 |  |  |  |  |  |  |
|  |  | *ω2* | 1 |  |  |  |  |  |  |  |  |  |  |
|  | M3 | *p_0_* | 0.67122 | *p_1_* | 0.29556 | *p_2_* | 0.03322 | M0 | vs | M3 | 470.063522 | 0 |  |
|  |  | *ω_0_* | 0.09389 | *ω_1_* | 0.75844 | *ω_2_* | 2.56812 |  |  |  |  |  |  |
|  | M7 | *p* | 0.39678 | *q* | 0.81327 |  |  |  |  |  |  |  |  |
|  | M8 | *p_0_* | 0.93332 | *p* | 0.45657 | *q* | 1.24013 | M7 | vs | M8 | 65.526302 | 0 |  |
|  |  | *p_1_* | 0.06668 | *ω* |  |  |  |  |  |  |  |  |  |
| *rps2* | M0 | *ω* | 0.20815 |  |  |  |  |  |  |  |  |  |  |
|  | M1a | *p_0_* | 0.80706 | *p_1_* | 0.19294 |  |  | M1a | vs | M2a | 0 | 1 |  |
|  | M2a | *p_0_* | 0.80706 | *p_1_* | 0.19294 | *p_2_* | 0 |  |  |  |  |  |  |
|  |  | *ω2* | 1 |  |  |  |  |  |  |  |  |  |  |
|  | M3 | *p_0_* | 0.498 | *p_1_* | 0.42185 | *p_2_* | 0.08015 | M0 | vs | M3 | 75.575716 | 0 |  |
|  |  | *ω_0_* | 0 | *ω_1_* | 0.31248 | *ω_2_* | 1.35399 |  |  |  |  |  |  |
|  | M7 | *p* | 0.21413 | *q* | 0.66021 |  |  |  |  |  |  |  |  |
|  | M8 | *p_0_* | 0.93651 | *p* | 0.26529 | *q* | 1.22943 | M7 | vs | M8 | 4.32E+00 | 0.115542 |  |
|  |  | *p_1_* | 0.06349 | *ω* | 1.46265 |  |  |  |  |  |  |  |  |
| *rps3* | M0 | *ω* | 0.22508 |  |  |  |  |  |  |  |  |  |  |
|  | M1a | *p_0_* | 0.82532 | *p_1_* | 0.17468 |  |  | M1a | vs | M2a | 6.00E-06 | 0.999997 |  |
|  | M2a | *p_0_* | 0.82532 | *p_1_* | 0.05798 | *p_2_* | 0.1167 |  |  |  |  |  |  |
|  |  | *ω2* | 1 |  |  |  |  |  |  |  |  |  |  |
|  | M3 | *p_0_* | 0.49146 | *p_1_* | 0.46877 | *p_2_* | 0.03977 | M0 | vs | M3 | 75.902042 | 0 |  |
|  |  | *ω_0_* | 0.02794 | *ω_1_* | 0.37244 | *ω_2_* | 2.04661 |  |  |  |  |  |  |
|  | M7 | *p* | 0.33206 | *q* | 0.86101 |  |  |  |  |  |  |  |  |
|  | M8 | *p_0_* | 0.96753 | *p* | 0.47088 | *q* | 1.65525 | M7 | vs | M8 | 12.057678 | 0.002408 |  |
|  |  | *p_1_* | 0.03247 | *ω* |  |  |  |  |  |  |  |  |  |
| *rps4* | M0 | *ω* | 0.23843 |  |  |  |  |  |  |  |  |  |  |
|  | M1a | *p_0_* | 0.79678 | *p_1_* | 0.20322 |  |  | M1a | vs | M2a | 0 | 1 |  |
|  | M2a | *p_0_* | 0.79678 | *p_1_* | 0.13167 | *p_2_* | 0.07155 |  |  |  |  |  |  |
|  |  | *ω2* | 1 |  |  |  |  |  |  |  |  |  |  |
|  | M3 | *p_0_* | 0.66991 | *p_1_* | 0.33009 | *p_2_* | 0 | M0 | vs | M3 | 13.988192 | 0.007333 |  |
|  |  | *ω_0_* | 0.04786 | *ω_1_* | 0.65979 | *ω_2_* | 1.52805 |  |  |  |  |  |  |
|  | M7 | *p* | 0.24793 | *q* | 0.7105 |  |  |  |  |  |  |  |  |
|  | M8 | *p_0_* | 0.99999 | *p* | 0.24793 | *q* | 0.71052 | M7 | vs | M8 | 1.80E-05 | 0.999991 |  |
|  |  | *p_1_* | 1E-05 | *ω* | 1 |  |  |  |  |  |  |  |  |
| *rps7* | M0 | *ω* | 0.44398 |  |  |  |  |  |  |  |  |  |  |
|  | M1a | *p_0_* | 0.52542 | *p_1_* | 0.47458 |  |  | M1a | vs | M2a | 1.80E-05 | 0.999991 |  |
|  | M2a | *p_0_* | 0.52584 | *p_1_* | 0.03822 | *p_2_* | 0.43594 |  |  |  |  |  |  |
|  |  | *ω2* | 1 |  |  |  |  |  |  |  |  |  |  |
|  | M3 | *p_0_* | 0.52585 | *p_1_* | 0.40417 | *p_2_* | 0.06998 | M0 | vs | M3 | 4.477814 | 0.345186 |  |
|  |  | *ω_0_* | 0 | *ω_1_* | 1.00182 | *ω_2_* | 1.00182 |  |  |  |  |  |  |
|  | M7 | *p* | 0.0712 | *q* | 0.08009 |  |  |  |  |  |  |  |  |
|  | M8 | *p_0_* | 0.52585 | *p* | 0.005 | *q* | 1.4156 | M7 | vs | M8 | 0.537926 | 0.764172 |  |
|  |  | *p_1_* | 0.47415 | *ω* | 1.00182 |  |  |  |  |  |  |  |  |
| *rps8* | M0 | *ω* | 0.25548 |  |  |  |  |  |  |  |  |  |  |
|  | M1a | *p_0_* | 0.77179 | *p_1_* | 0.22821 |  |  | M1a | vs | M2a | 0 | 1 |  |
|  | M2a | *p_0_* | 0.77179 | *p_1_* | 0.15877 | *p_2_* | 0.06944 |  |  |  |  |  |  |
|  |  | *ω2* | 1 |  |  |  |  |  |  |  |  |  |  |
|  | M3 | *p_0_* | 0.65116 | *p_1_* | 0.22256 | *p_2_* | 0.12628 | M0 | vs | M3 | 53.501596 | 0 |  |
|  |  | *ω_0_* | 0.0677 | *ω_1_* | 0.42351 | *ω_2_* | 1.20364 |  |  |  |  |  |  |
|  | M7 | *p* | 0.26408 | *q* | 0.6129 |  |  |  |  |  |  |  |  |
|  | M8 | *p_0_* | 0.86165 | *p* | 0.6929 | *q* | 3.61242 | M7 | vs | M8 | 4.214874 | 0.121549 |  |
|  |  | *p_1_* | 0.13835 | *ω* |  |  |  |  |  |  |  |  |  |
| *rps11* | M0 | *ω* | 0.1971 |  |  |  |  |  |  |  |  |  |  |
|  | M1a | *p_0_* | 0.9124 | *p_1_* | 0.0876 |  |  | M1a | vs | M2a | 0.441356 | 0.801975 |  |
|  | M2a | *p_0_* | 0.92509 | *p_1_* | 0.05671 | *p_2_* | 0.0182 |  |  |  |  |  |  |
|  |  | *ω2* | 1 |  |  |  |  |  |  |  |  |  |  |
|  | M3 | *p_0_* | 0.23992 | *p_1_* | 0.71649 | *p_2_* | 0.04359 | M0 | vs | M3 | 27.532978 | 1.55E-05 |  |
|  |  | *ω_0_* | 0 | *ω_1_* | 0.21192 | *ω_2_* | 1.53986 |  |  |  |  |  |  |
|  | M7 | *p* | 0.53968 | *q* | 1.78935 |  |  |  |  |  |  |  |  |
|  | M8 | *p_0_* | 0.96128 | *p* | 1.14537 | *q* | 5.46569 | M7 | vs | M8 | 7.770434 | 0.020543 |  |
|  |  | *p_1_* | 0.03872 | *ω* | 1.61576 |  |  |  |  |  |  |  |  |
| *rps12* | M0 | *ω* | 0.69433 |  |  |  |  |  |  |  |  |  | 23 K |
|  | M1a | *p_0_* | 0.82154 | *p_1_* | 0.17846 |  |  | M1a | vs | M2a | 45.502512 | 0 |  |
|  | M2a | *p_0_* | 0.95621 | *p_1_* | 0 | *p_2_* | 0.04379 |  |  |  |  |  |  |
|  |  | *ω2* | 1 |  |  |  |  |  |  |  |  |  |  |
|  | M3 | *p_0_* | 0 | *p_1_* | 0.95621 | *p_2_* | 0.04379 | M0 | vs | M3 | 65.68356 | 0 |  |
|  |  | *ω_0_* | 0 | *ω_1_* | 0.5032 | *ω_2_* | 999 |  |  |  |  |  |  |
|  | M7 | *p* | 0.0582 | *q* | 0.07181 |  |  |  |  |  |  |  |  |
|  | M8 | *p_0_* | 0.95623 | *p* | 0.06645 | *q* | 0.06251 | M7 | vs | M8 | 48.164398 | 0 |  |
|  |  | *p_1_* | 0.04377 | *ω* | 999 |  |  |  |  |  |  |  |  |
| *rps14* | M0 | *ω* | 0.33629 |  |  |  |  |  |  |  |  |  |  |
|  | M1a | *p_0_* | 0.68801 | *p_1_* | 0.31199 |  |  | M1a | vs | M2a | 5.273798 | 0.071583 |  |
|  | M2a | *p_0_* | 0.7325 | *p_1_* | 0 | *p_2_* | 0.2675 |  |  |  |  |  |  |
|  |  | *ω2* | 1 |  |  |  |  |  |  |  |  |  |  |
|  | M3 | *p_0_* | 0.7325 | *p_1_* | 0.05019 | *p_2_* | 0.21731 | M0 | vs | M3 | 55.400222 | 0 |  |
|  |  | *ω_0_* | 0.0511 | *ω_1_* | 1.39025 | *ω_2_* | 1.39025 |  |  |  |  |  |  |
|  | M7 | *p* | 0.04638 | *q* | 0.06097 |  |  |  |  |  |  |  |  |
|  | M8 | *p_0_* | 0.73329 | *p* | 5.52982 | *q* | 99 | M7 | vs | M8 | 1.9753 | 0.372451 |  |
|  |  | *p_1_* | 0.26671 | *ω* | 1.39255 |  |  |  |  |  |  |  |  |
| *rps15* | M0 | *ω* | 0.40171 |  |  |  |  |  |  |  |  |  |  |
|  | M1a | *p_0_* | 0.66616 | *p_1_* | 0.33384 |  |  | M1a | vs | M2a | 0 | 1 |  |
|  | M2a | *p_0_* | 0.66616 | *p_1_* | 0.20913 | *p_2_* | 0.12471 |  |  |  |  |  |  |
|  |  | *ω2* | 1 |  |  |  |  |  |  |  |  |  |  |
|  | M3 | *p_0_* | 0.66064 | *p_1_* | 0.31234 | *p_2_* | 0.02702 | M0 | vs | M3 | 29.336688 | 6.68E-06 |  |
|  |  | *ω_0_* | 0.11902 | *ω_1_* | 0.97938 | *ω_2_* | 0.97938 |  |  |  |  |  |  |
|  | M7 | *p* | 0.26459 | *q* | 0.40797 |  |  |  |  |  |  |  |  |
|  | M8 | *p_0_* | 0.66805 | *p* | 14.0427 | *q* | 99 | M7 | vs | M8 | 1.260538 | 0.532449 |  |
|  |  | *p_1_* | 0.33195 | *ω* | 1 |  |  |  |  |  |  |  |  |
| *rps16* | M0 | *ω* | 0.39292 |  |  |  |  |  |  |  |  |  |  |
|  | M1a | *p_0_* | 0.60084 | *p_1_* | 0.39916 |  |  | M1a | vs | M2a | 4.205822 | 0.1221 |  |
|  | M2a | *p_0_* | 0.57886 | *p_1_* | 0.40399 | *p_2_* | 0.01715 |  |  |  |  |  |  |
|  |  | *ω2* | 1 |  |  |  |  |  |  |  |  |  |  |
|  | M3 | *p_0_* | 0.57483 | *p_1_* | 0.4079 | *p_2_* | 0.01727 | M0 | vs | M3 | 41.371646 | 2.3E-08 |  |
|  |  | *ω_0_* | 0.04831 | *ω_1_* | 0.98036 | *ω_2_* | 5.34161 |  |  |  |  |  |  |
|  | M7 | *p* | 0.06517 | *q* | 0.07981 |  |  |  |  |  |  |  |  |
|  | M8 | *p_0_* | 0.98197 | *p* | 0.04958 | *q* | 0.05762 | M7 | vs | M8 | 4.10742 | 0.128258 |  |
|  |  | *p_1_* | 0.01803 | *ω* |  |  |  |  |  |  |  |  |  |
| *rps18* | M0 | *ω* | 0.26714 |  |  |  |  |  |  |  |  |  |  |
|  | M1a | *p_0_* | 0.71564 | *p_1_* | 0.28436 |  |  | M1a | vs | M2a | 1.107264 | 0.574858 |  |
|  | M2a | *p_0_* | 0.72144 | *p_1_* | 0.23037 | *p_2_* | 0.04819 |  |  |  |  |  |  |
|  |  | *ω2* | 1 |  |  |  |  |  |  |  |  |  |  |
|  | M3 | *p_0_* | 0.54045 | *p_1_* | 0.29093 | *p_2_* | 0.16862 | M0 | vs | M3 | 64.755824 | 0 |  |
|  |  | *ω_0_* | 0.02906 | *ω_1_* | 0.33373 | *ω_2_* | 1.57144 |  |  |  |  |  |  |
|  | M7 | *p* | 0.06997 | *q* | 0.0915 |  |  |  |  |  |  |  |  |
|  | M8 | *p_0_* | 0.82933 | *p* | 0.36911 | *q* | 2.0425 | M7 | vs | M8 | 3.688924 | 0.15811 |  |
|  |  | *p_1_* | 0.17067 | *ω* | 1.56356 |  |  |  |  |  |  |  |  |
| *rps19* | M0 | *ω* | 0.32291 |  |  |  |  |  |  |  |  |  |  |
|  | M1a | *p_0_* | 0.74937 | *p_1_* | 0.25063 |  |  | M1a | vs | M2a | 0.39201 | 0.822008 |  |
|  | M2a | *p_0_* | 0.82318 | *p_1_* | 0 | *p_2_* | 0.17682 |  |  |  |  |  |  |
|  |  | *ω2* | 1 |  |  |  |  |  |  |  |  |  |  |
|  | M3 | *p_0_* | 0.82318 | *p_1_* | 0.10168 | *p_2_* | 0.07514 | M0 | vs | M3 | 10.00866 | 0.040282 |  |
|  |  | *ω_0_* | 0.14019 | *ω_1_* | 1.41087 | *ω_2_* | 1.41087 |  |  |  |  |  |  |
|  | M7 | *p* | 0.1902 | *q* | 0.34787 |  |  |  |  |  |  |  |  |
|  | M8 | *p_0_* | 0.82473 | *p* | 16.44729 | *q* | 99 | M7 | vs | M8 | 1.029464 | 0.597661 |  |
|  |  | *p_1_* | 0.17527 | *ω* | 1.41571 |  |  |  |  |  |  |  |  |
| *ycf2* | M0 | *ω* | 1.01284 |  |  |  |  |  |  |  |  |  | 54 W, 157 I, 159 I, 190 S, 212 L, 229 F, 398 L, 430 H, 447 N, 472 Y, 473 H, 474 I, 475 P, 493 D, 506 Y, 509 R, 538 A, 547 K, 548 F, 611 L, 630 Q, 681 Y, 683 P, 693 L, 755 F, 843 R, 882 K, 883 D, 911 S, 915 P, 1001 F, 1063 R, 1071 P, 1088 S, 1091 T, 1189 G, 1190 S, 1215 R, 1295 T, 1351 C, 1408 L, 1410 D, 1413 L, 1419 T, 1420 E, 1421 L, 1423 T, 1427 T, 1432 R, 1436 T, 1467 S, 1469 G, 1502 N, 1504 F, 1576 T, 1589 D, 1654 F, 1674 H, 1739 Y, 1740 E, 1741 S, 1746 A, 1754 L, 1778 W, 1783 Y, 1790 Q, 1794 S, 1818 R, 1830 F, 1859 P, 1860 P, 1912 G |
|  | M1a | *p_0_* | 0.40446 | *p_1_* | 0.59554 |  |  | M1a | vs | M2a | 271.198842 | 0 |  |
|  | M2a | *p_0_* | 0.9151 | *p_1_* | 0 | *p_2_* | 0.0849 |  |  |  |  |  |  |
|  |  | *ω2* | 1 |  |  |  |  |  |  |  |  |  |  |
|  | M3 | *p_0_* | 0.84308 | *p_1_* | 0.15264 | *p_2_* | 0.00428 | M0 | vs | M3 | 567.445126 | 0 |  |
|  |  | *ω_0_* | 0.56001 | *ω_1_* | 3.90501 | *ω_2_* | 40.65299 |  |  |  |  |  |  |
|  | M7 | *p* | 1.06612 | *q* | 0.73664 |  |  |  |  |  |  |  |  |
|  | M8 | *p_0_* | 0.92662 | *p* | 6.1214 | *q* | 2.8972 | M7 | vs | M8 | 370.223108 | 0 |  |
|  |  | *p_1_* | 0.07338 | *ω* | 6.74446 |  |  |  |  |  |  |  |  |
| *ycf3* | M0 | *ω* | 0.12773 |  |  |  |  |  |  |  |  |  |  |
|  | M1a | *p_0_* | 0.91384 | *p_1_* | 0.08616 |  |  | M1a | vs | M2a | 0.014574 | 0.992739 |  |
|  | M2a | *p_0_* | 0.92077 | *p_1_* | 0 | *p_2_* | 0.07923 |  |  |  |  |  |  |
|  |  | *ω2* | 1 |  |  |  |  |  |  |  |  |  |  |
|  | M3 | *p_0_* | 0.09502 | *p_1_* | 0.82574 | *p_2_* | 0.07924 | M0 | vs | M3 | 12.456324 | 0.014262 |  |
|  |  | *ω_0_* | 0.07193 | *ω_1_* | 0.07193 | *ω_2_* | 1.07715 |  |  |  |  |  |  |
|  | M7 | *p* | 0.16444 | *q* | 0.82376 |  |  |  |  |  |  |  |  |
|  | M8 | *p_0_* | 0.92261 | *p* | 7.91724 | *q* | 99 | M7 | vs | M8 | 1.879346 | 0.390756 |  |
|  |  | *p_1_* | 0.07739 | *ω* | 1.08841 |  |  |  |  |  |  |  |  |
| *ycf4* | M0 | *ω* | 0.29672 |  |  |  |  |  |  |  |  |  |  |
|  | M1a | *p_0_* | 0.78358 | *p_1_* | 0.21642 |  |  | M1a | vs | M2a | 1.64778 | 0.438722 |  |
|  | M2a | *p_0_* | 0.83838 | *p_1_* | 0.01951 | *p_2_* | 0.14211 |  |  |  |  |  |  |
|  |  | *ω2* | 1 |  |  |  |  |  |  |  |  |  |  |
|  | M3 | *p_0_* | 0.28823 | *p_1_* | 0.58935 | *p_2_* | 0.12242 | M0 | vs | M3 | 59.279322 | 0 |  |
|  |  | *ω_0_* | 0 | *ω_1_* | 0.23634 | *ω_2_* | 1.5458 |  |  |  |  |  |  |
|  | M7 | *p* | 0.20561 | *q* | 0.40712 |  |  |  |  |  |  |  |  |
|  | M8 | *p_0_* | 0.87882 | *p* | 0.83854 | *q* | 3.99019 | M7 | vs | M8 | 6.790198 | 0.033537 |  |
|  |  | *p_1_* | 0.12118 | *ω* | 1.54197 |  |  |  |  |  |  |  |  |
|  | | | | | | | | | | | | | |
